# Innervate Commercial Fabrics with Spirally‐Layered Iontronic Fibrous Sensors Toward Dual‐Functional Smart Garments

**DOI:** 10.1002/advs.202402767

**Published:** 2024-07-02

**Authors:** Xiaodong Wu, Qi Liu, Lifei Zheng, Sijian Lin, Yiqun Zhang, Yangyang Song, Zhuqing Wang

**Affiliations:** ^1^ School of Mechanical Engineering Sichuan University Chengdu 610065 China; ^2^ Med+X Center for Manufacturing West China Hospital Sichuan University Chengdu 610041 China

**Keywords:** electronic textile, fibrous sensor, mechanical sensing, smart garment, thermal sensing

## Abstract

Electronic fabrics exhibit desirable breathability, wearing comfort, and easy integration with garments. However, surficial deposition of electronically functional materials/compounds onto fabric substrates would consequentially alter their intrinsic properties (e.g., softness, permeability, biocompatibility, etc.). To address this issue, here, a strategy to innervate arbitrary commercial fabrics with unique spirally‐layered iontronic fibrous (SLIF) sensors is presented to realize both mechanical and thermal sensing functionalities without sacrificing the intrinsic fabric properties. The mechanical sensing function is realized via mechanically regulating the interfacial ionic supercapacitance between two perpendicular SLIF sensors, while the thermal sensing function is achieved based on thermally modulating the intrinsic ionic impedance in a single SLIF sensor. The resultant SLIF sensor‐innervated electronic fabrics exhibit high mechanical sensitivity of 81 N^−1^, superior thermal sensitivity of 34,400 Ω °C^−1^, and more importantly, greatly minimized mutual interference between the two sensing functions. As demonstrations, various smart garments are developed for the precise monitoring of diverse human physiological signals. Moreover, artificial intelligence‐assisted object recognition with high‐accuracy (97.8%) is demonstrated with a SLIF sensor‐innervated smart glove. This work opens up a new path toward the facile construction of versatile smart garments for wearable healthcare, human‐machine interfaces, and the Internet of Things.

## Graphical abstract

A universal strategy is presented to innervate arbitrary commercial fabrics with unique spirally‐layered iontronic fibrous (SLIF) sensors to realize both mechanical and thermal sensing functionalities without sacrificing the intrinsic fabric properties. With this strategy, a wide spectrum of smart garments, including smart mask, wrist band, smart glove, etc., are successfully demonstrated for vital signal monitoring and object recognition.

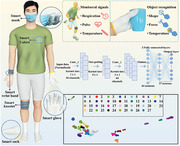

## 1 Introduction

Wearable sensing technology has made remarkable progress in recent year benefiting from the advancement in material innovations,^[^ ^1^ ^]^ structure engineering,^[^ ^2^ ^]^ and systemic device integrations.^[^ ^3^ ^]^ Nevertheless, the essential components of most wearable sensors are composed of rigid inorganic materials or stiff polymeric materials,^[^ ^4^ ^]^ compromising their conformality, wearing comfort, and breathability.^[^ ^5^ ^]^ To mitigate these issues, electronic textiles (e‐textiles) have been proposed by integrating specific sensing functionalities into fibrous or fabric materials.^[^ ^6^ ^]^ Inheriting the nature of textiles, e‐textiles exhibit unique features such as lightness, flexibility, breathability, and easy integration with commercial garments.^[^ ^7^ ^]^ Additionally, e‐textiles possess good scalability and customization flexibility.^[^ ^8^ ^]^ These characteristics make e‐textiles promising candidates for various applications such as health and exercise monitoring,^[^ ^9^ ^]^ smart home,^[^ ^10^ ^]^ and human‐computer interaction,^[^ ^11^ ^]^ exhibiting great potential to profoundly transform our routines in the future.

Significant progress has been achieved for constructing e‐textiles in the past decades. In general, there are two strategies to fabricate e‐textiles with desired functionalities. The first strategy is to uniformly deposit electronically functional materials or compounds on fabric substrates through coating,^[^ ^12^ ^]^ dyeing,^[^ ^13^ ^]^ evaporation,^[^ ^14^ ^]^ chemical treatment^[^ ^15^ ^]^ and so on.^[^ ^16^ ^]^ Additionally, in order to save functional materials and to improve the designability of the e‐textiles, selective functionalization of fabric substrates at targeted areas can be achieved via printing,^[^ ^17^ ^]^ mask‐assisted deposition,^[^ ^18^ ^]^ lamination,^[^ ^19^ ^]^ and so on.^[^ ^20^ ^]^ Despite the effectiveness of these two surficial deposition strategies to construct functional e‐textiles, two essential issues are usually inevitable, as discussed in Table S1 (Supporting Information). On one hand, depositing functional materials on fabric substrates will consequentially alter the basic properties (e.g., softness, permeability, biocompatibility, etc.) of the original fabrics.^[^ ^16b^ ^]^ On the other hand, the deposited functional materials on fabric substrates suffer from exfoliation problems during wearing, abrasion, or washing, leading to deteriorating functionality with time and potential biotoxicity.^[^ ^11b^ ^]^

Alternatively, integration of preformed functional sensing fibrous sensors into fabric substrates via weaving or sewing methods provides another approach to construct e‐textiles with desirable functions.^[^ ^21^ ^]^ Compared with the surficial coating strategy, this approach endows the fabrics with specific sensing functions and, in the meanwhile, maintains their original characteristics (e.g., softness, permeability, wearing comfort, etc., see Table S1, Supporting Information for more discussion).^[^ ^22^ ^]^ Nevertheless, up to now, the integrated functional fibrous sensors normally possess only one sensing function (e.g., strain sensing, pressure sensing, temperature sensing, etc.) due to their confined fibrous form factors,^[^ ^23^ ^]^ significantly limiting their application scenarios. Some multimodal fibrous sensors have been proposed to detect different stimulations (e.g., pressure, strain, flexion, etc.).^[^ ^24^ ^]^ However, the mutual interference/crosstalk between different sensing modes would deteriorate the sensing accuracy and reliability of specific targets. Moreover, the multiple sensing modes cannot be operated simultaneously. Hence, expanding the sensing capability and functionality and, at the meantime, minimizing the mutual interference/crosstalk between different sensing modes pose two main roadblocks for constructing multifunctional and versatile e‐textiles.

**Figure 1:**
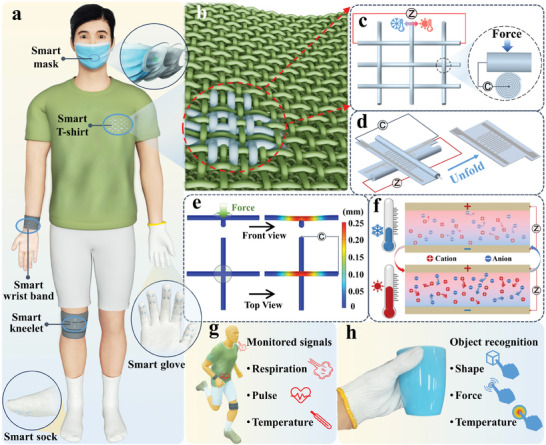
Design concept of innervating commercial fabrics with SLIF sensors for smart garments. a) Schematic illustrates various smart garments innervated with SLIF sensors. b) Illustration showing the integration of SLIF sensors in fabric substrates. c) 3D configurations and measuring methods of mechanical and thermal sensing units based on SLIF sensors. d) Illustrations showing the internal spiral structure of the SLIF sensors. e) Finite element analysis (FEA) showing the intersectional contact area variations between two perpendicular SLIF sensors under an external force. f) Diagram illustrating the thermal sensing mechanism of the SLIF sensors. g,h) Application scenarios of the SLIF sensor innervated smart garments for monitoring physiological signals and object recognition.

Motivated by the aforementioned challenges, here, we propose to innervate arbitrary commercial fabrics with a new paradigm of spirally‐layered iontronic fibrous (SLIF) sensors for simultaneous but selective detection of both mechanical and thermal stimulations. The SLIF sensors are composed of spirally‐structured interdigitated silver (Ag) electrodes and thermoplastic polyurethane (TPU)/ionic liquid (IL) iontronic sensing layers. After integrating the SLIF sensors into commercial fabrics (**Figure** **1**a,b), external mechanical stimulations could prominently regulate the interfacial ionic supercapacitance between two perpendicular SLIF sensors (Figure 1c–e), while external thermal stimulations would modulate intrinsic ionic impedance in a single SLIF sensor (Figure 1c,d,f), enabling to resolve both mechanical and thermal stimulations. More importantly, the mutual interference between the mechanical and thermal sensing modes can be significantly minimized based on rational configuration engineering and sensing mechanism design. The mechanical sensing units of the SLIF sensors exhibit high mechanical sensitivity up to 81 N^−1^, fast response/recovery time (66/79 ms), low detection limit (76 mg), and excellent cycling stability (10 000 cycles). The thermal sensing units shows thermal sensitivity up to 34400 Ω °C^−1^ and a low minimum temperature detection of 0.1 °C. Notably, innervating commercial fabrics with the SLIF sensors does not compromise the basic properties (e.g., softness, permeability, wearing comfort, etc.) of the fabric substrates. As demonstrations, various SLIF sensor‐innervated wearable smart garments are developed to continuously monitor a variety of physiological vital signals (e.g., respiration rate, pulse rate, body temperature, etc.). Besides, the SLIF sensors are integrated into smart gloves for grasp perception and object recognition. By combining the mechanical/thermal dual sensing functions and rational artificial intelligence (AI) algorithm, high‐accuracy (97.8%) object recognition can be achieved within 27 objects of different shapes, weights, and temperatures. This study opens up a new path to extend the functionality and versatility of e‐textiles as well as smart garments without sacrificing their intrinsic characteristics.

## 2 Results and Discussion

### 2.1 Design Concept of Innervating Commercial Fabrics with SLIF Sensors

Figure 1a,b shows the schematics of innervating commercial fabrics with the SLIF sensors for constructing diverse smart garments. Via sewing or weaving methods, the SLIF sensors could be easily integrated into arbitrary fabric substrates (Figure S1, Supporting Information), exhibiting good flexibility and adaptability in constructing different smart garments. After innervation of the SLIF sensors, the original form factor and basic properties of the fabric substrates will not be altered significantly.

The 3D configuration and cross‐section morphology of the SLIF sensors are illustrated in Figure 1c,d. The SLIF sensors consist of spirally‐layered TPU/IL ionic composites and spirally‐structured interdigitated electrodes. In the fabric substrates, the SLIF sensors are orthogonally integrated. The intersections of two perpendicular sensors form mechanical sensing units, based on mechanically regulated ionic supercapacitance variations between the two SLIF sensors. As revealed from Figure 1e, the contact area of the intersections between two sensors would increase under mechanical stimulations, leading to a remarkable upswing in the ionic supercapacitance measured between the two SLIF sensors. This mechanical sensing mechanism based on interfacial ionic supercapacitance variations exhibits much higher sensitivity compared to traditional capacitive sensing devices.^[^ ^25^ ^]^

Thermal sensing function is realized in a single SLIF sensor based on thermally regulated ionic impedance variations of the TPU/IL ionic composites (Figure 1f). Lower temperatures result in slower ion migration and higher ionic impedance, and vice versa. The impedance signal is measured by the spirally‐structured interdigitated electrodes embedded inside the SLIF sensors. This iontronic thermal sensing mechanism also shows much higher sensitivity when compared to conventional resistive sensing devices.^[^ ^24c^ ^]^

By innervating the dual‐functional SLIF sensors into commercial garments, a wide spectrum of human physiological signals can be monitored conveniently and efficiently (Figure 1g). Also, high accuracy recognition of objects with various shapes, weights, and temperatures can be realized by using the SLIF sensor innervated smart gloves (Figure 1h).

### 2.2 Fabrication and Characterization of SLIF Sensors

**Figure 2:**
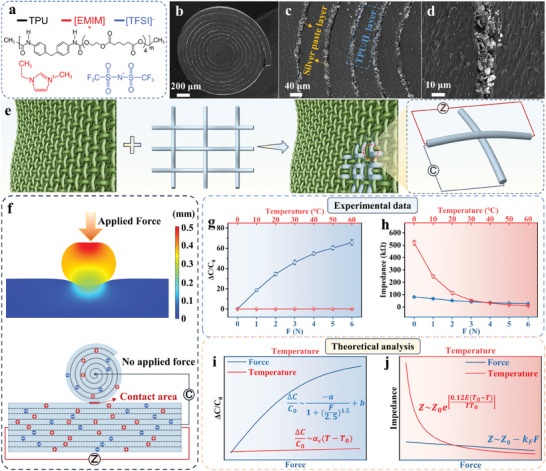
Fabrication and characterization of SLIF sensors. a) Material chemistry of the SLIF sensors. b–d) Cross‐sectional SEM images of the SLIF sensors with different magnifications. e) Diagrams of innervating fabric substrates with SLIF sensors to develop functional e‐textiles. f) Simulation result of intersectional contact area changes between two perpendicular SLIF sensors before and after applying a force. g) Experimental results of the relative capacitance changes (*ΔC*/*C_0_*) of the mechanical sensing units under different forces and temperatures. h) Experimental results of the ionic impedance (*Z*) of the thermal sensing units under different forces and temperatures. i) Theoretical analysis of the relative capacitance changes of the mechanical sensing units under different forces and temperatures. j) Theoretical analysis of the ionic impedance of the thermal sensing units under different forces and temperatures.

**Figure** **2**a presents the material chemical properties of components in SLIF sensors. TPU is selected as the soft and elastic matrix, and organic ionic liquid is employed as the ion source for iontronic mechanical and thermal sensations. With good solubility of the ionic liquid in the TPU matrix,^[^ ^26^ ^]^ the SLIF sensors do not suffer leakage of ionic liquid under different harsh conditions (Figure S2, Supporting Information). The fabrication process of the SLIF sensors is illustrated in Figure S3 (Supporting Information). Interdigitated electrodes were printed on TPU/IL films with elastic silver paste, and the final SLIF sensors with a spirally‐layered structure were produced by rolling. More details about fabrication process can be found in the Experimental Section and Supporting Information.

The cross‐section morphology of the SLIF sensors is presented in Figure 2b. A spirally layered structure can be clearly observed, with alternate TPU/IL layers (dark and thick line) and silver electrode layer (bright and thin line). Under high resolution (Figure 2c,d), silver flakes in the electrode layers could be well distinguished. This unique spirally‐layered and alternately segregated structure possesses two distinct merits. First, the conductive silver flakes are selectively distributed rather than randomly dispersed in the TPU/IL matrix, which allows to acquire highly conductive networks using only a small amount of silver flakes and also help to maintain good mechanical softness of the SLIF sensors. Second, the silver electrode layers are embedded into the TPU/IL matrix rather than attached onto the SLIF sensors, possessing superior mechanical robustness and abrasion resistance.

Figure 2e illustrates the innervation of fabric substrates with the SLIF sensors. With good flexibility and softness (Figure S4, Supporting Information), the SLIF sensors can be easily integrated into arbitrary fabric substrates with self‐defined patterns. This innervation process can endow the fabrics with both mechanical and thermal sensing functions, and also maximally preserve the original properties of the fabric substrates. As shown in Figure S5 (Supporting Information), the mechanical and thermal sensing performance can be regulated by the IL content and diameters of the SLIF sensors (see Figure S5, Supporting Information for more discussion).

For multifunctional sensing devices, the interference and crosstalk between different sensing modes cause problems for reliable detection of each single stimulation. In this work, we alleviate this issue by rational configuration engineering and sensing mechanism design. Specifically, the interfacial supercapacitance variation between two perpendicular SLIF sensors was employed for mechanical sensing, while the intrinsic ionic impedance variation in a single SLIF sensor was used for thermal sensing. Such configuration and mechanism designs allow to significantly reducing the interference between these two sensing modes.

Both experimental verification and theoretical analysis were conducted to demonstrate the abovementioned assumptions. Figure 2f depicts the simulation result of intersectional geometry change between two perpendicular SLIF sensors. Without an external force applied on the intersection, the intersectional contact area is very small. Applying a force could remarkably increase the intersectional contact area from the finite element analysis, resulting in the corresponding increase of the interfacial ionic capacitance. As presented in Figure 2g, the relative capacitance change (Δ*C*/*C_0_*) of the intersectional SLIF sensors was measured under different mechanical forces and different temperatures. The experiment results show that the force variation causes much higher change in Δ*C*/*C_0_* than temperature variation. The variation of Δ*C*/*C_0_* from 0 to 60 °C under a force of 1 N is negligible (Δ*C*/*C_0_* ≈0.2), which is significantly smaller than the relative capacitance change caused by applying a mechanical force of 6 N (Δ*C*/*C₀* ≈66), revealing the selective sensitivity of such sensing configuration to mechanical stimulations rather than thermal stimuli.

On the other hand, the impact of mechanical force on the thermal sensing performance is also evaluated. As shown in Figure 2h, when increasing the applied force from 0 to 6 N at 25 °C, the ionic impedance (*Z*) shows only 52 kΩ variation, which is much smaller than the impedance variation when elevating the temperature from 0 to 60 °C (with 510 kΩ variation). These results indicate the selective sensitivity of the thermal sensing units to thermal stimulations rather than mechanical stimuli.

In addition to experimental demonstrations, we conduct theoretical analysis to further verify our assumptions. For the mechanical sensing units, the ionic capacitance of the mechanical sensing units can be expressed as *C* = *C_s_* ∙ *A*, where *C_s_* denotes the unit area capacitance and *A* is the intersectional contact area.^[^ ^27^ ^]^ *C_s_* can be regarded as a constant in a given system. Therefore, the interfacial capacitance depends mainly on the contact area. Initially, the intersectional contact area increases rapidly with the applied force. Then, the growth rate of the contact area gets saturated. Based on the above analysis, we derived a relationship equation (Figure S6, Supporting Information) between force (*F*) and relative capacitance change of the SLIF sensors by nonlinear fitting of the experimental results:

(1) $$\frac{\Delta C}{C_{0}}\;∼\;\frac{-a}{1+{\left(\frac{F}{2.5}\right)}^{1.5}}+b$$

where *a* and *b* are constants related to the mechanical sensing system (*a* < *b*). From Equation 1, it is reflected that Δ*C*/*C_0_* increases with the *F* rapidly, but finally get saturated as *F* increases further.

Based on the aforementioned conclusion of *C* = *C_s_* ∙ *A*, the effect of temperature variation (*ΔT*) on Δ*C*/*C_0_* is reflected in the change of unit area capacitance (*C_s_*) and intersectional contact area (*A*). On one hand, the variation of intersectional contact area resulting from Δ*T* is negligible. On the other hand, the unit area capacitance would change via the change in ionic migration and dielectric constant of ionic composites due to Δ*T*,^[^ ^28^ ^]^ while relevant researches concerning the temperature dependence of ionic capacitance showing that the variation of unit area capacitance resulting from Δ*T* is usually less than 50%.^[^ ^29^ ^]^ The effects of temperature (*T*) on the Δ*C*/*C_0_* can be expressed as follows:

(2) $$\frac{\Delta C}{C_{0}}\;∼\;\alpha_{c}\left(T-T_{0}\right)$$

where α_*c*_ is the temperature coefficient of ionic capacitance and proved to be a small value (0 < *α_c_* ≪ 1).^[^ ^29^ ^]^ *T_0_* is the initial temperature value. Based on Equations 1 and 2, it can be seen that the relative capacitance change caused by force variation is significantly greater than that caused by temperature change, as shown in Figure 2i, which is consistent with the experiment results.

For the thermal sensing units, the temperature sensing function is realized based on measuring the ionic impedance or ionic conductance in a single SLIF sensor. The relationship between temperature (*T*) and the ionic conductivity (*σ*) of the SLIF sensors can be evaluated through the Arrhenius equation:

(3) $$\sigma \;=\sigma_{0}\;e^{-\left(\frac{E_{a}}{K_{B}T}\right)}$$

where *σ_0_* is the pre‐exponential parameter, *E_a_* is the activation energy and *K_B_* denotes the Boltzmann constant. Based on the above equation, the approximate relationship between the ionic impedance and *T* can be derived as:

(4) $$Z\;∼\;Z_{0}e^{\left[\frac{0.12E\left(T_{0}-T\right)}{TT_{0}}\right]}$$

where *Z_0_* stands for the initial impedance value, *E* denotes activation energy.^[^ ^30^ ^]^ It can be seen that temperature variation has a prominent influence on the ionic impedance of the SLIF sensors. Finally, the impedance variation would gradually get saturated with *T* increases further, which is consistent with the experiment results.

Applying a mechanical force would also affect the geometry of the SLIF sensors and might have an influence on the measured ionic impedance, but such effect is very limited. Specifically, under mechanical pressing, the SLIF sensors would be compressed with a slight reduction in the diameter of the SLIF sensors. As a result, the measured ionic impedance decreases slightly. The negative correlation between the applied force and the measured impedance value of the SLIF sensors can be expressed with the following relationship:

(5) $$Z\;∼\;Z_{0}-k_{F}F$$

where *k_F_* could be regarded as a small constant representing the coefficient of force on reduced impedance (0 < *k_F_* ≪ 1). Based on Equations 4 and 5, it can be reflected that the ionic impedance variation resulting from temperature change is much greater than that caused by external force. Therefore, the ionic impedance variation of SLIF sensors is mainly dominated by the temperature change rather than mechanical stimulations (Figure 2j), which also agree well with the experiment results. The above results reveal the significantly minimized interference between mechanical and thermal sensing processes, ensuring the reliable detection of mechanical and temperature stimuli, respectively.

### 2.3 Mechanical and Thermal Sensing Performance of SLIF Sensors

**Figure 3:**
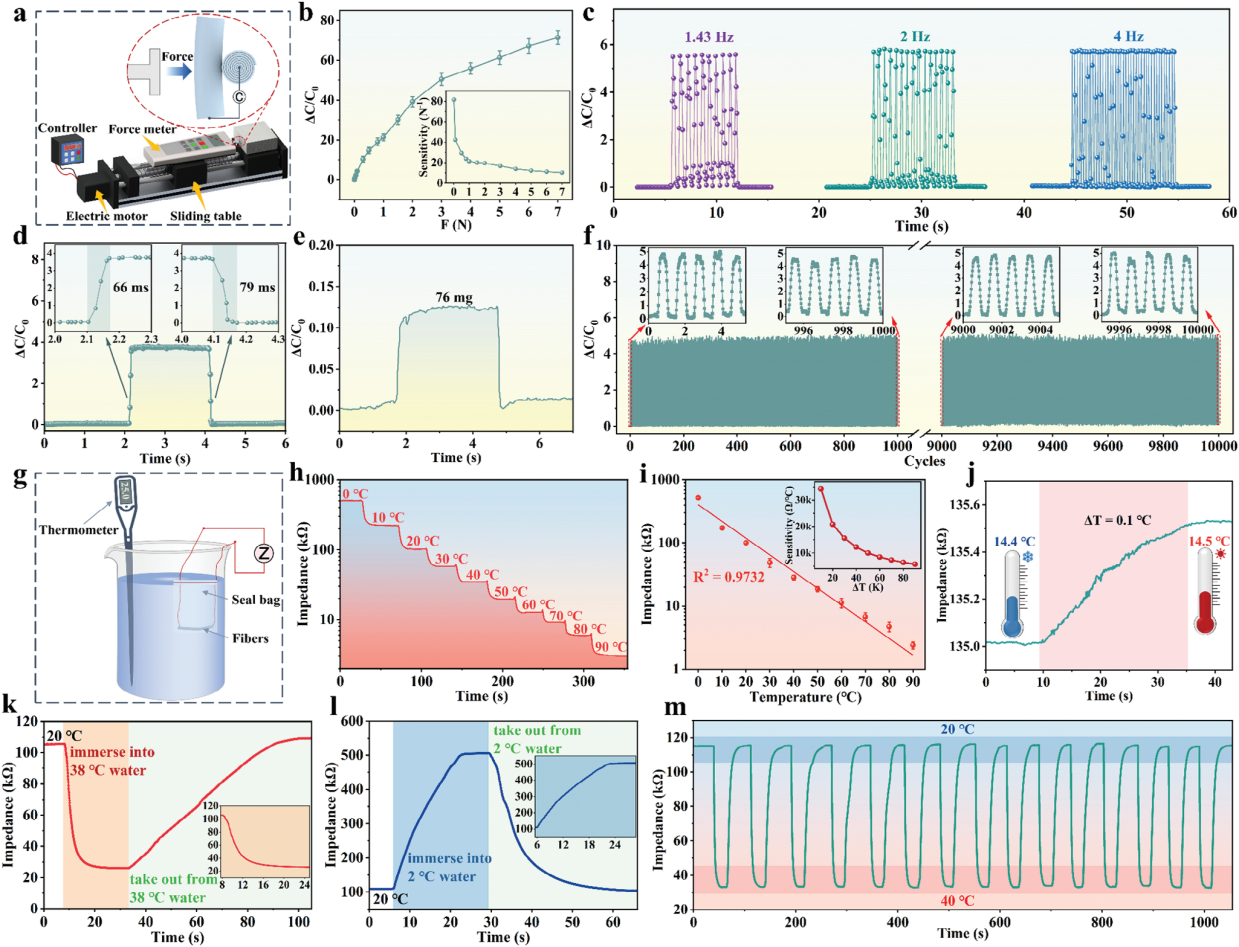
Mechanical and thermal sensing performance of SLIF sensors. a) Mechanical testing setup of the mechanical sensing units. b) Relative capacitance change (Δ*C*/*C_0_*) curves of the mechanical sensing units under different forces. The inset shows the variation in mechanical sensitivity. c) Response behaviors of the mechanical sensing units under forces of different frequencies. d) Response and recovery time of the mechanical sensing units. e) Response behavior of the mechanical sensing units when loading and removing of a 76 mg object. f) Repeatability test of the mechanical sensing units for 10 000 cycles under 2 N force. g) Thermal testing setup of the thermal sensing units. h) Impedance change of the thermal sensing units when the temperature elevates from 0 to 90 °C with 10 °C interval. i) Log plot of the impedances of the thermal sensing units under different temperatures. The inset shows the thermal sensitivity. j) The minimum temperature detectability (0.1 °C) of the SLIF sensors. k,l) Response curves of the thermal sensing units (under 20 °C room temperature) when immersed into the 38 °C warm water baths k) and 2 °C cold water bath l), followed by the removing the sensors from the water baths. m) Cyclic response curves of the thermal sensing units with the temperature alternatively changing from 20 to 40 °C.

The mechanical and thermal sensing properties of the SLIF sensors are systematically investigated. The measurement setup for mechanical sensing performance is shown in **Figure** **3**a. The initial capacitance of the mechanical sensing units is ≈0.8–1.2 pF. The response behavior of the mechanical sensing units under different applied forces is shown in Figure 3b. Increasing the applied force (*F*) gives rise to higher relative capacitance change (Δ*C*/*C_0_*). The mechanical sensitivity, which is defined as Δ*C*/*C_0_*/Δ*F*, exhibits a maximum value of 81 N^−1^ in the beginning and gradually decreases with the applied force. The force‐induced dramatic signal variations endow the SLIF sensors with good resistance to external disturbance, including wind, wire jitter, and parasitic capacitance (Figure S7, Supporting Information). When applying a continuous force from 0 to 5 N in a stepwise manner, the mechanical sensing units show a corresponding stepwise response (Figure S8, Supporting Information), indicating the capacity of the sensors for continuous monitoring of static or slowly varying mechanical stimuli.

Figure 3c and Figure S9 (Supporting Information) present the frequency‐dependent sensing behaviors of the mechanical sensing units from 1.43 to 10 Hz, revealing that dynamic mechanical stimuli can also be resolved. The response time and recovery time are measured to be ≈66 ms and ≈79 ms at 0.2 N force, respectively (Figure 3d). The response and recovery times of the mechanical sensing units have a positive correlation with the applied force (see Figure S10, Supporting Information for more discussion). The SLIF sensors also have a low detection limit. As revealed from Figure 3f, a subtle mechanical stimulus by applying a 76 mg object can be well recognized by the sensors. Moreover, the mechanical sensing units shows high stability in a cyclic test for 10 000 cycles (Figure 3f), confirming the excellent durability and reliability of SLIF sensors.

The thermal sensing behaviors of the SLIF sensors were investigated using water baths of controlled temperature (Figure 3g). The impedance of the thermal sensing units in the temperature range of 0–90 °C is shown in Figure 3h and Figure S11 (Supporting Information). It shows that the thermal sensors can detect different temperatures over a wide range in a continuous and reliable manner. The measured ionic impedance of the sensors shows remarkable decrease (i.e., two orders of magnitude) when elevating the temperature from 0 to 90 °C. The thermal sensitivity can be defined as the impedance change caused by temperature variation, which is expressed as *ΔZ*/Δ*T* = (*Z – Z_0_*)/Δ*T* with a unit of Ω °C^−1^. From Figure 3i, it is revealed that a high thermal sensitivity up to 34 400 Ω °C^−1^ can be achieved, which is much higher than that of conventional resistive temperature sensors.^[^ ^31^ ^]^ Notably, the minimum temperature detection limit of the SLIF sensors can be as low as 0.1 °C (Figure 3j), making these sensors well qualified for precise temperature measurement.

When placing the thermal sensors into a warm water bath of 38 °C from an initial temperature of 20 °C, the signal output of the thermal sensors gradually decreased until reaching a steady state (Figure 3k). After being removed from the warm water, the signal output recovered to the initial value gradually. In the contrary, when the thermal sensors were immersed into a cold water bath of 2 °C, the signal output increased gradually and reached a steady state after a while (Figure 3l). Upon removal from the cold water, the signal output returned to the initial state. As shown in Figure 3m, a highly stable and repeatable signal variation is observed when alternatively subjecting the sensors to 20 and 40 °C, demonstrating the repeatability and reliability of the SLIF sensors.

The effect of humidity change on the sensing performance of the SLIF sensors is also evaluated. The results show that the SLIF sensors are not highly sensitive to external humidity (Figure S12, Supporting Information). Furthermore, the SLIF sensors exhibit superior mechanical robustness and abrasion resistance, including bending (Figures S13 and S14, Supporting Information), washing (Figure S15, Supporting Information), treading (Figure S16, Supporting Information). This is due to that the silver electrode layers are embedded into the TPU/IL matrix rather than attached to the SLIF sensors.

### 2.4 Smart Mask and Wrist Band for Physiological Signal Monitoring

**Figure 4:**
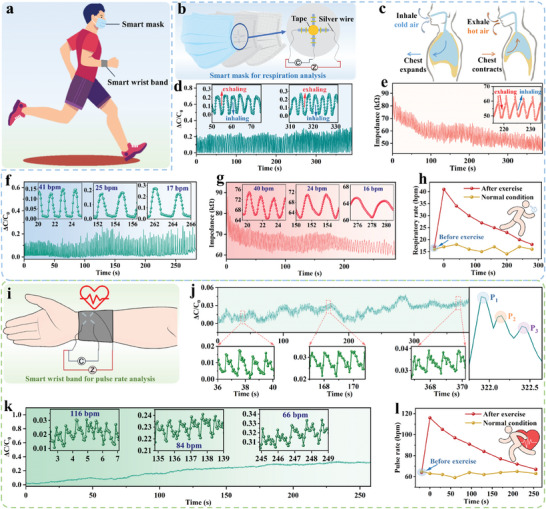
Human vital signal monitoring based on the SLIF sensor‐innervated smart garments. a) Schematic showing monitoring physiological signal of the subject based on the SLIF sensor‐innervated smart mask and wrist band. b) Schematic showing that the SLIF sensors are integrated into the filter layer of a mask to construct smart masks. c) Schematic diagram of human inhalation and exhalation process. d,e) Continuously recorded respiration signals with the smart mask by the mechanical sensing mode d) and thermal sensing mode e). f,g) Continuously recorded respiration signals of the subject after taking exercise by the mechanical sensing mode f) and thermal sensing mode g). h) Variation in respiratory rates of the subject before (normal condition) and after exercise. i) Schematics showing a wrist band innervated with the SLIF sensors. j,k) Continuously recorded pulse signals of the subject before j) and after exercise k) with the smart wrist band. l) Variation in pulse rates of the subject before (normal condition) and after exercise.

The proposed SLIF sensors feature two advantages: 1) easy integration into arbitrary garments while maintaining their intrinsic wearing comfort; 2) superior sensing performance in term of both versatility and capability. These distinct merits enable the SLIF sensors to construct diverse smart garments for wearable healthcare and human‐machine interfacing. As demonstrations, we conducted the real‐time monitoring of human physiological signals based on the SLIF sensor‐innervated smart garments (**Figure** **4**a).

As depicted in Figure 4b, a dual‐functional smart mask is developed for respiration monitoring by integrating two perpendicular SLIF sensors into a medical mask. The smart mask features two sensing modes (i.e., force sensing mode and temperature sensing mode). Both of the two sensing modes can be utilized for high‐accuracy respiration analysis. The reliability of the independent mechanical sensing capabilities is evaluated (Figure S17, Supporting Information).

A healthy subject (male, 23 years old) was selected to verify such dual mode sensing capabilities. As shown in Figure 4d,e, the respiratory rhythm can be well detected by either the mechanical or the thermal sensing modes. As illustrated in Figure 4c, the exhalation process is associated to an increase in both temperature and force, leading to a decrease in both impedance signal and an increase in capacitance signal. On the contrary, the inhalation process gives rise to the increase in impedance signal and decrease in capacitance signal. In addition to respiratory rhythm, via temperature calibration (Figure S18, Supporting Information), the practical temperature variation during the respiration process can also be extracted as shown in Figure S19 (Supporting Information), which provides more information to evaluate the respiratory conditions of the subject.

The respiration conditions of the subject before and after exercise were also evaluated. As given in Figure 4f,g, the respiratory condition variations (e.g., respiratory rate) can be reflected from both of the two sensing modes. Notably, the respiratory rates calculated from the two sensing modes are always kept consistent during the whole exercise process (Figure 4f,g). The respiratory rate variation is extracted from the whole process, as presented in Figure 4h. When the subject is kept at rest, the calculated respiratory rate remains nearly constant with ≈15 bpm on average. In contrast, after the subject did 5 min of exercise, the respiratory rate ramps up to 41 bpm. After the subject stops the exercise, the calculated respiratory rate exhibits a downtrend and gradually decreases to a stable state with time. In addition, the respiratory process can also be monitored using a SLIF sensor‐innervated smart elastic belt (Figure S20, Supporting Information). These results verify the dual functionality of the SLIF sensors for reliably monitoring the respiratory conditions.

The pulse signal serves as another vital sign of the human body and reflects the dynamic cardiovascular status. Pulse signal could also be monitored and analyzed with the SLIF sensor‐innervated wrist band. As shown in Figure 4i, two perpendicular SLIF sensors are integrated into a commercial wrist band, by which the arterial blood pressure variations can be monitored in real‐time. The recorded arterial waveforms (Figure 4j) have three characteristic peaks, including the percussion wave (P_1_ wave), tidal wave (P_2_ wave), and diastolic wave (P_3_ wave).

Pulse rate can be extracted from the pulse signals. As demonstrations, the pulse rate variation of the subject was monitored before and after taking exercise for 5 min. As presented in Figure 4k, the pulse rate of the subject ramps to 116 bpm after taking exercise. After stopping exercise, the pulse rate gradually declines to a normal condition (Figure 4l). Moreover, via careful calibration (Figure S21, Supporting Information), the developed smart wrist band is qualified for detecting the skin surface temperature, as demonstrated in Figure S22 (Supporting Information).

Furthermore, we have also innervated garments with the SLIF sensors to construct smart T‐shirt, smart kneelet and smart sock. A variety of human signals could be monitored, including the skin temperature (Figure S23, Supporting Information), bending of knee (Figure S24, Supporting Information), and walking movement (Figure S25, Supporting Information). These results demonstrate the potential applications of the SLIF sensors for constructing wearable smart garments for personal healthcare.

### 2.5 Smart Glove for AI‐Assisted Object Recognition

**Figure 5:**
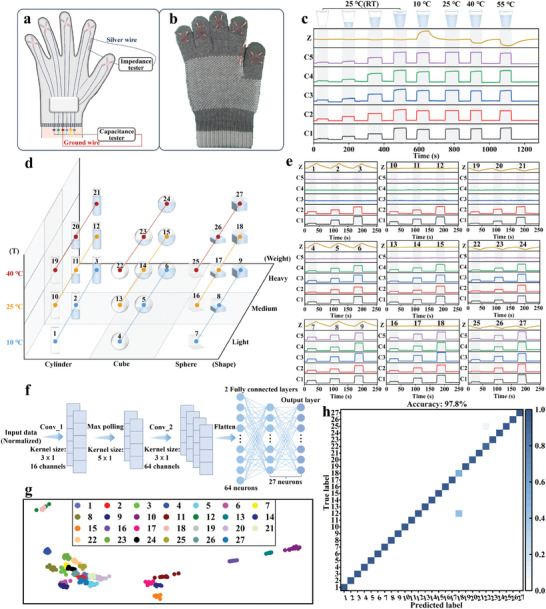
SLIF sensor innervated smart glove for grasp perception and object recognition. a) Schematic illustration showing a SLIF sensor‐innervated smart glove. b) Photograph of the SLIF sensor‐innervated smart glove, with two perpendicular SLIF sensors innervated to each finger respectively and an additional single SLIF sensor innervated to the index finger. c) Response signals recorded by the five mechanical sensing units and one thermal sensing unit when grasping the cups of different weight and temperature with a critical force. d) Illustration showing 27 objects with different shapes, weights, and temperatures for object recognition test. e) Response signals recorded by the five mechanical sensing units and one thermal sensing unit when grasping the 27 different objects with a critical force. f) Schematic diagram showing the artificial neural network used for object recognition. g) T‐distributed stochastic neighbor embedding visualization of clustered data. h) Confusion matrix of the 27 tested objects, showing a high recognition accuracy of 97.8%.

Equipped with both mechanical and thermal sensing capabilities, the SLIF sensors exhibit promising potential to create touch sensation functions for prosthetic and robotic systems. Here, we demonstrate SLIF sensor‐innervated smart gloves for versatile touch sensation and object recognition. As presented in **Figure** **5**a,b, perpendicular SLIF sensors are innervated to each finger of the gloves to form both mechanical and thermal sensing units. The force variation and temperature change during grasping different objects could be resolved with these smart gloves. As a preliminary demonstration, dixie cups with different weights of water and different temperatures were used as the objects. The subject who wore the smart gloves was asked to grasp the dixie cups with a critical force that can hold the cups without sliding and does not crumb the cups. Five mechanical sensing signals on each finger and one thermal sensing signal on the index were recorded. As shown in Figure 5c, the critical force used for holding the cups increases with the amount of the water in the cups, and the temperature can also be reflected from the impedance signals. These results reveal that the smart glove can be used to differentiate objects with different weights and temperatures.

To systematically demonstrate the touch sensation functionality of the SLIF sensor‐innervated smart gloves, 27 hollow objects with different shapes (e.g., cube, cylinder, and sphere), different weights (i.e., filling objects with different amounts of water), and different temperatures (i.e., 10, 25, and 40 °C) were selected as the targeted objects (Figure 5d). Critical grasp movements that can just pick the objects up without sliding as mentioned above were conducted. Figure 5e presents all situations of the response signals from the five mechanical sensing units and one thermal sensing unit. It shows that, when grasping objects with different shapes, different finger combinations were used due to the habit of the subject. Specifically, as depicted in Figure S26 (Supporting Information), the cylinder grasping activated two mechanical sensing units, the cube grasping activated four, and the sphere grasping activated five sensing units. In addition, the weight and temperature of the objects could be reflected from the intensity of the maximum capacitance output and impedance output during the grasps. Heavier objects and higher temperatures give rise to higher capacitance and lower impedance outputs respectively. By comparing and analyzing the five mechanical sensing signals and one thermal sensing signal, the shape, weight, and temperature of the objects can be recognized.

Due to the complexity in processing the multichannel signals, here, we employed an artificial intelligence (AI) algorithm to realize high‐accuracy object recognition with our smart gloves. An artificial neural network (ANN) model was developed and trained, which consists of two convolution layers, a max pooling layer, and two fully‐connected layers (Figure 5f). A total of 40 sets of data for each object were collected, and 70% of the data was used for training purposes, while the remaining sets were reserved for testing. The t‐distributed stochastic neighbor embedding (t‐SNE) technique was used for dimensionality reduction and data visualization. The visualization results, as shown in Figure 5g, demonstrate that the datasets corresponding to different objects are well clustered together, indicating the distinguishable characteristics of each object. This ANN model exhibits an average classification accuracy of 97.8%, as depicted in the confusion matrix of Figure 5h, revealing the desirable object recognition ability of the smart gloves. These results highlight the promising potential of SLIF sensor‐innervated smart gloves in human‐computer interactions, such as efficient AR/VR controls and dexterous object manipulations.

## 3 Conclusion

In summary, we have presented a universal strategy to innervate arbitrarily commercial fabrics with iontronic SLIF sensors that feature unique spirally‐structured interdigitated electrodes embedded into spirally‐layered TPU/IL ionic composites. The proposed SLIF sensors feature highly sensitive but selective responses to mechanical and thermal stimulations respectively. The e‐textiles innervated with the SLIF sensors exhibit high mechanical sensing performance (including high sensitivity of 81 N^−1^, low detection limit of 76 mg, fast response/recovery of 66/79 ms, and good cyclic stability over 10 000 cycles) and outstanding thermal sensing capabilities (e.g., thermal sensitivity up to 34400 Ω °C^−1^, minimum detectable temperature change of 0.1 °C, etc.). Moreover, the interference between the mechanical sensing and thermal sensing processes could be significantly minimized based on the proposed structure and sensing mechanism designs. A variety of smart garments with good comfortability, breathability, and versatility have been demonstrated by innervating the SLIF sensors into commercial fabrics. Physiological vital signals, including body temperature, pulse rate, respiratory signal, and various body motions, can be precisely and continuously monitored with these smart garments. Furthermore, high‐accuracy object recognition (97.8%) has been successfully realized with SLIF sensor‐innervated gloves using artificial intelligence algorithms. This work shows great potential to expand the functionalities of e‐textiles and smart garments without sacrificing their original characteristics, which could find promising applications in smart wearables, healthcare devices, human‐machine interfaces, and the Internet of Things.

## 4 Experimental Section

### Materials

Thermoplastic polyurethanes (TPU) powders (Desmopan 6005) were supplied by Bayer AG (Germany). N, N‐Dimethylformamide (DMF) solvent and ionic liquid (IL) of 1‐Ethyl‐3‐Methylimidazolium Bis (Trifluoromethylsulfonyl) Imide ([EMIM]^+^[TFSI]^−^) was provided from Adamas Reagent co., Ltd. (China). The Polytetrafluoroethylene (PTFE) molds were provided by YiXing Experimental Equipment Co., Ltd (China). Elastic conductive silver (Ag) paste (CI‐1036) was provided by Engineered Materials Solutions, Inc. (USA).

### Fabrication of TPU/IL Substrate film

First, TPU powders were dissolved into DMF solvent at a mass ratio of 1.5:10 to form TPU solution. 10 wt% IL with respect to the weight of TPU was added into the TPU solution. Then, the TPU/IL mixture solution was magnetically stirred at 80 °C for 1 h to achieve uniform mixture and the TPU/IL‐DMF solution was obtained. The TPU/IL films were prepared by the solution casting method. The well‐mixed TPU/IL‐DMF solution was casted into a flat PTFE mold, followed by drying in an air circulated oven at 70 °C for 1 h. After drying the DMF solvent, the TPU/IL films were carefully peeled off from PTFE mold with tweezers. The eventual TPU/IL films with good softness, elasticity, and self‐adhesiveness can be obtained.

### Printing Ag Interdigitated Electrodes on TPU/IL Substrate

The elastic interdigital electrodes were printed on the TPU/IL films by a screen‐printing method. A digitally designed screen‐mesh with interdigital electrode patterns was put on the TPU/IL films, and elastic conductive silver paste was applied onto the screen‐mesh. Using a rubber squeegee, the paste was smoothly scraped across the screen mesh as the squeegee moved from one end to the other. After screen printing, the printed interdigital electrodes on the TPU/IL substrate were dried in the oven at 80 °C for 30 min to cure the silver paste and to improve its electrical conductivity and mechanical robustness.

### Preparation of SLIF Sensors

The unnecessary portion of the TPU/IL films with printed electrode patterns was cut off, leaving the interdigital electrodes area only. Then, the TPU/IL films with printed electrode patterns were manually rolled up by applying a shearing force, finally into a fibrous shape. The fibrous shape of the TPU/IL films after rolling up can be well maintained due to the self‐adhesive property of the TPU/IL composite. Subsequently, the SLIF sensors with spirally‐layered interdigital electrodes were cured at a temperature of 80 °C for 1 h. During this process, good fusion and bonding could occur between the spirals TPU/IL layers, giving rise to stable and robust SLIF sensors, with the spirally‐layered silver electrode layers were firmly adhered onto the two adjacent TPU/IL layers. Thin and conductive yarns (i.e., silver‐coated nylon yarns) were adhered onto the pads of the interdigital electrodes with silver paste to facilitate the signal acquisition process.

### Simulation of Mechanical Sensing Units

In order to investigate the assumed mechanical sensing principle based on mechanically regulated contact area change of the intersections between two perpendicular SLIF sensors, relevant simulation was conducted using the finite element analysis (FEA) method. The geometric model of two perpendicular SLIF sensors was simplified as two vertically‐crossed cylinders with the diameter of 1 mm. The sensor was regarded as a homogeneous isotropic rubbery material with Young's modulus of 15 MPa and Poisson's ratio of 0.47. Boundary load condition was applied above the intersection of the two cylinders, static constraint condition was added below the bottom cylinder, and contact pair condition was applied to the contact surfaces of cylinders. This model was employed to simulate the contact area change of the intersections between two perpendicular SLIF sensors when applying forces to the mechanical sensing units.

### Artificial Intelligence Algorithm for Object Recognition

An artificial neural network was trained to recognize the grasped objects. For 27 different objects, a total of 27 × 40 sets of data were collected, and each object had 1800 features as the input of the ANN model. To enhance the generalization capability of model, the dataset was first preprocessed by using a Min‐Max normalization method. The ANN model consists of two convolution layers, one max pooling layer and two fully‐connected layers. The first and second convolution layers have 16 and 64 channels respectively, the size of the convolution kernel is 3 × 1 and the activation function is the Rectified Linear Unit (ReLU) function. For the kernel of the max pooling layer, the size is 5 × 1. The first fully connected layer consists of 64 neurons with the ReLU function as the activation function. The subsequent fully connected layer comprises 27 neurons, employing the Softmax function as the activation function. Seventy percent of the dataset were randomly divided into training sets, tenfold cross‐validation method was used for validation, and the remaining thirty percent of the dataset were taken as test sets. The loss function was the Cross‐Entropy function, and the stochastic gradient descent algorithm was adopted as algorithm optimization. The initial learning rate was set as 0.1 and 200 epochs have been trained.

### Characterization and Measurement

The cross‐sectional scanning electron microscopy (SEM) observations of the SLIF sensors were conducted using a scanning electron microscope (ZEISS Sigma 300, Germany). A custom‐made mechanical measurement setup consists of a stepping motor, a programmable motor controller (KH‐01, China), a sliding stage, and a digital force meter (Handpi‐HLD, China) was employed to conduct the mechanical sensing performance tests of the SLIF sensors, where the force meter placed on the sliding table was used to apply the targeted force on sensors. A folded piece of paper weighing 76 mg (2 cm × 1.6 cm) was quickly placed on the intersection of two perpendicular SLIF sensors for a few seconds and then promptly removed it using tweezers to test the force detection limit of the SLIF sensors. Thermal sensing performance tests were conducted in a water bath with controlled temperature. The capacitance and impedance signal outputs of the SLIF sensors were collected on a high‐speed LCR meter (TH2817B, China) at the frequency of 100 kHz. For the application scenario of object grasping with the smart gloves, the multi‐channel capacitance signals were collected using a transformer comprehensive tester (ZX2789‐I109, China). During human physiological vital signals and motions monitoring, the part of the SLIF sensors contacting the skin was covered with an insulating plastic wrap to eliminate the body capacity effect. All human subjects participating in the tests involving the human body agreed to all tests and the picture in the manuscript, and all tests were approved by the Scientific Ethical Committee of the School of Mechanical Engineering, Sichuan University.

### Ethical Clearence

The experiments involving human subjects have been performed with the full, informed consent of the volunteers.

## Conflict of Interest

The authors declare no conflict of interest.

## Supporting information

Supporting Information

## Data Availability

Data sharing is not applicable to this article as no new data were created or analyzed in this study.
