# Supplementary material for: Innervate Commercial Fabrics with Spirally‐Layered Iontronic Fibrous Sensors Toward Dual‐Functional Smart Garments
Source: Adv Sci (Weinh). 2024 Jul 2;11(33):2402767. doi: 10.1002/advs.202402767 (PMC11434216; doi:10.1002/advs.202402767)
Supplement: Supplementary file 1 — Supporting Information [file ADVS-11-2402767-s001.docx]

**Supporting Information**

Innervate commercial fabrics with spirally-layered iontronic fibrous sensors towards dual-functional smart garments

Xiaodong Wu^†^, Qi Liu^†^, Lifei Zheng, Sijian Lin, Yiqun Zhang, Yangyang Song, and Zhuqing Wang*

X. Wu, Q. Liu, L. Zheng, S. Lin, Y. Zhang, Y. Song, Z. Wang

School of Mechanical Engineering, Sichuan University, Chengdu 610065, China

Z. Wang

Med+X Center for Manufacturing, West China Hospital, Sichuan University, Chengdu 610041, China

*E-mail address: wzhuqing@scu.edu.cn

†These authors have equal contribution to this work.

**Table S1. Comparison of three strategies** **to construct e-textiles with desired functionalities.**

| **Strategy** | **Effect on pristine textiles** | **Refs** |
| --- | --- | --- |
| 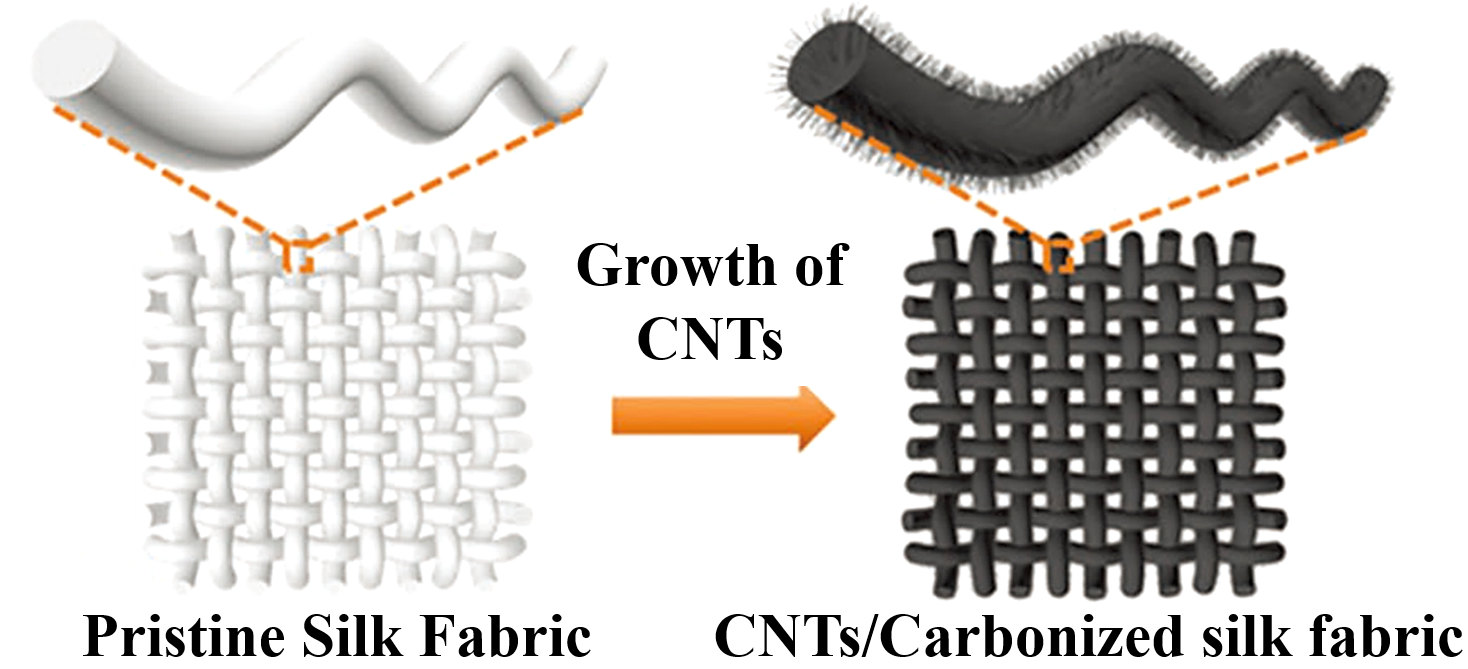**Uniform coating or deposition** | - Completely alter the original properties of the textile substrates - Exfoliation of functional materials and possible biotoxicity | ^[1]^ |
| 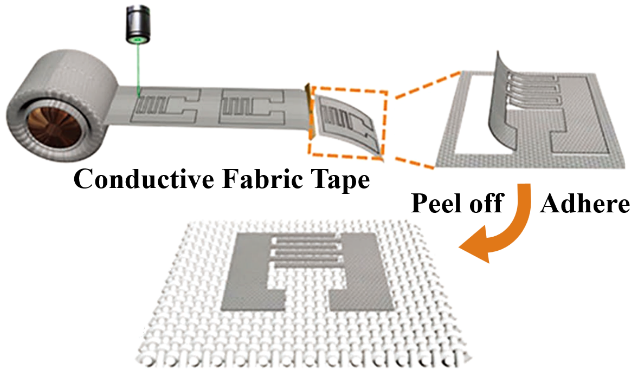**Selective coating or deposition** | - Partly alter the original properties of the textile substrates - Exfoliation of functional materials and possible biotoxicity | ^[1]^ |
| 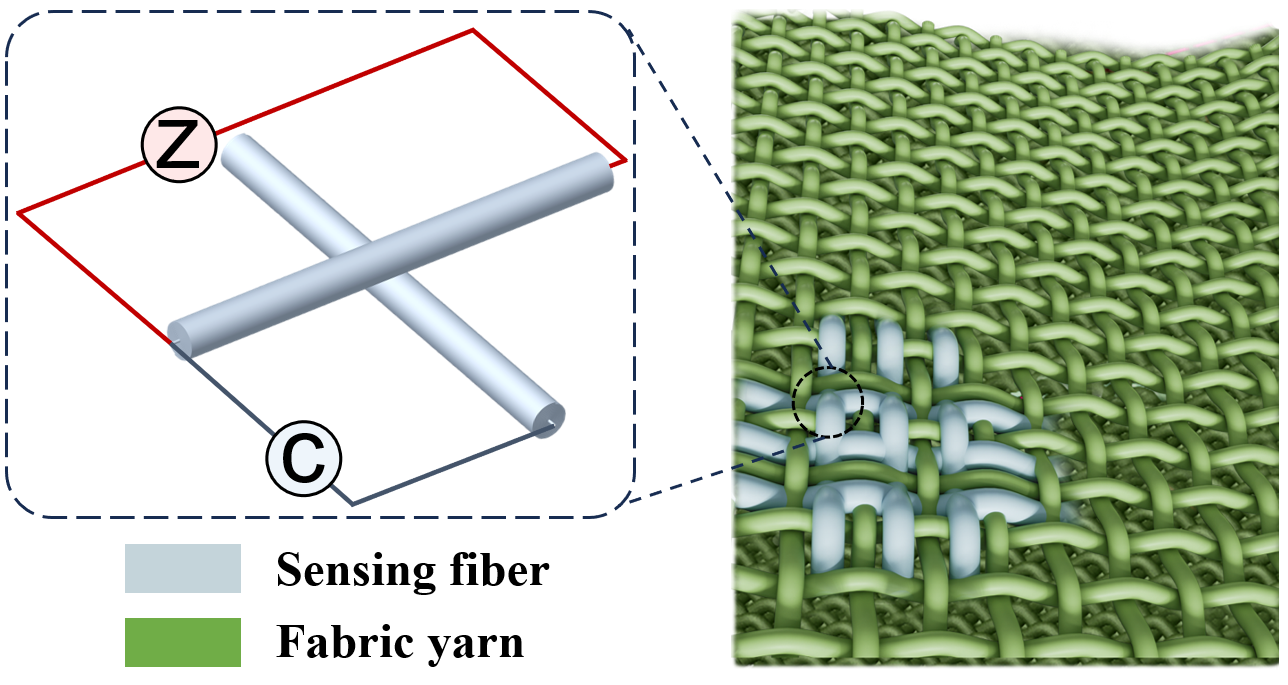**Innervating fabrics with functional fibers** | - Minimized influence on the properties of the textile substrates - Mechanical robustness and stability due to the spiral structure design | This work |

For the first strategy to construct e-textiles, specific functional materials or compounds is uniformly deposited on the entire textile substrates, thus to acquire desired functionalities.^[2]^ For instance, oxidative chemical vapor deposition of poly(3,4-thylenedioxythiophene) (PEDOT) is employed to form uniform conducting polymer layers on various commercial fabrics to fabricate wearable sensors.^[3]^

In contrast to uniform functionalization, the second strategy enables the targeted selective functionalization of fabric substrates, which can save the functional materials and improve the designability of e-textiles. For example, a programmable dual-regime spray is utilized to enable the direct custom writing of functional nanoparticles into fabrics for desired e-textiles.^[4]^

However, the aforementioned two surficial deposition strategies would completely or partly alter the inherent properties of the pristine textile, which deteriorates the comfort, lightness and biocompatibility of e-textiles.^[5]^ More importantly, the functional materials deposited on fabric substrates are prone to exfoliation issues during applications due to wearing, abrasion, and washing, leading to deteriorating functionality with time and potential biotoxicity.^[6]^

The third strategy is to innervate fabric substrates with preformed functional sensing fibrous sensors to construct e-textiles with desirable functions. For example, Luo et al. developed the coaxial conductive fibers coated with a piezoresistive nanocomposite through an automated coating technique, and large-scale sensing textiles can be obtained by introducing these functional fibers into fabrics via digital machine knitting.^[7]^ Besides, Gao et al. proposed the conductive microfibers with exceptional strain performance by incorporating carbon nanotubes (CNTs) into polyurethane (PU) fibers, and the microfibers are integrated into garments to construct a wearable smart healthcare system.^[8]^ Compared with the surficial coating strategies, this approach endows the fabrics with specific sensing functions while retaining their inherent features (e.g., softness, permeability, wearing comfort, etc.). Moreover, innervating fabrics with functional fibrous sensors enabling the provision of specific functions at the fiber level, and enhances the customizability and extendibility of e-textiles.^[9]^


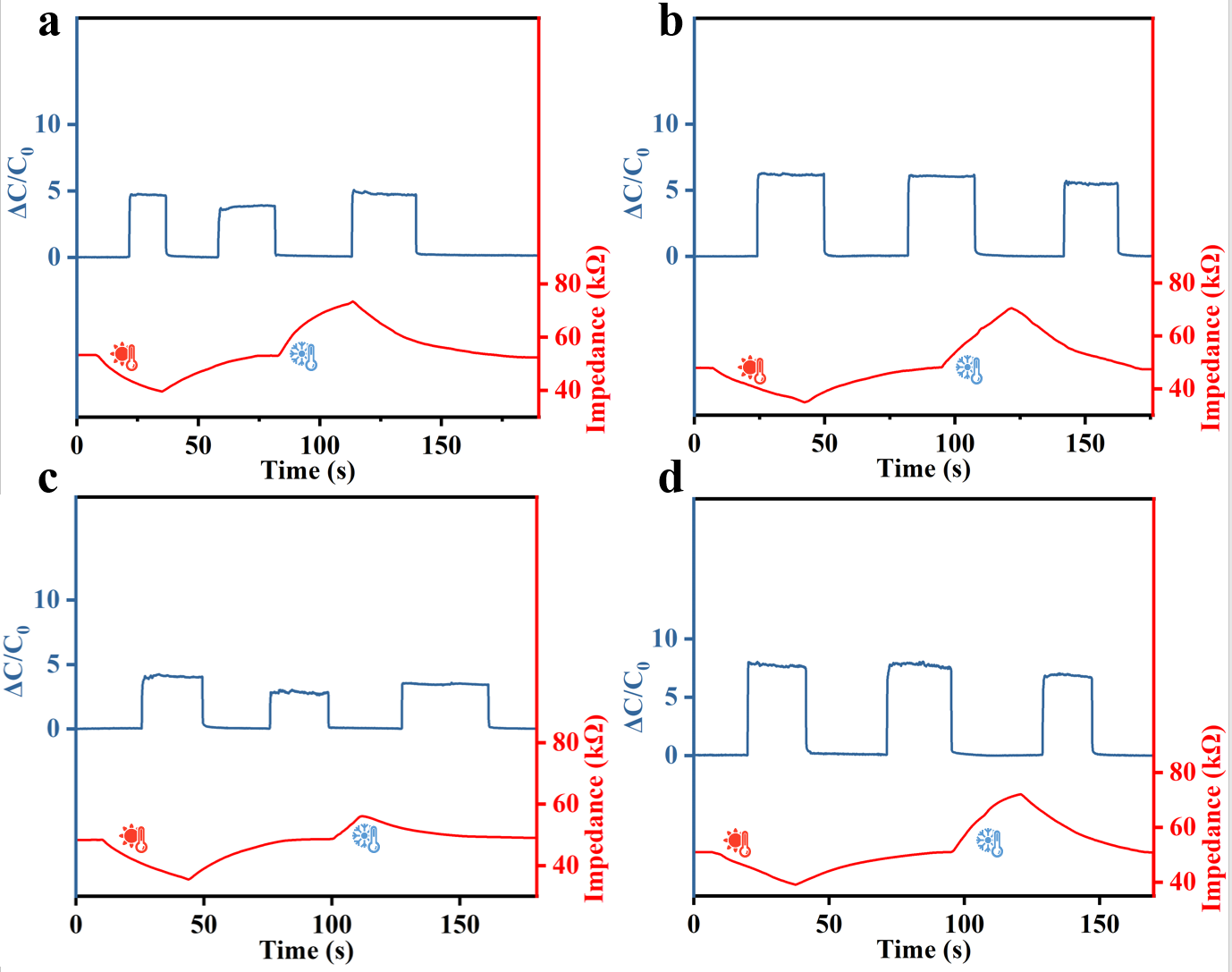


**Figure S1.** **Innervating different commercial fabrics with SLIF sensors for construct smart garments.** a) cotton, b) polyester, c) nylon, d) spandex.

Despite of the difference in the fabric substrates, the mechanical and thermal sensing functions of the SLIF sensor-innervated fabrics are not affected significantly, demonstrating the universality of innervating arbitrary fabrics with the proposed SLIF sensors.


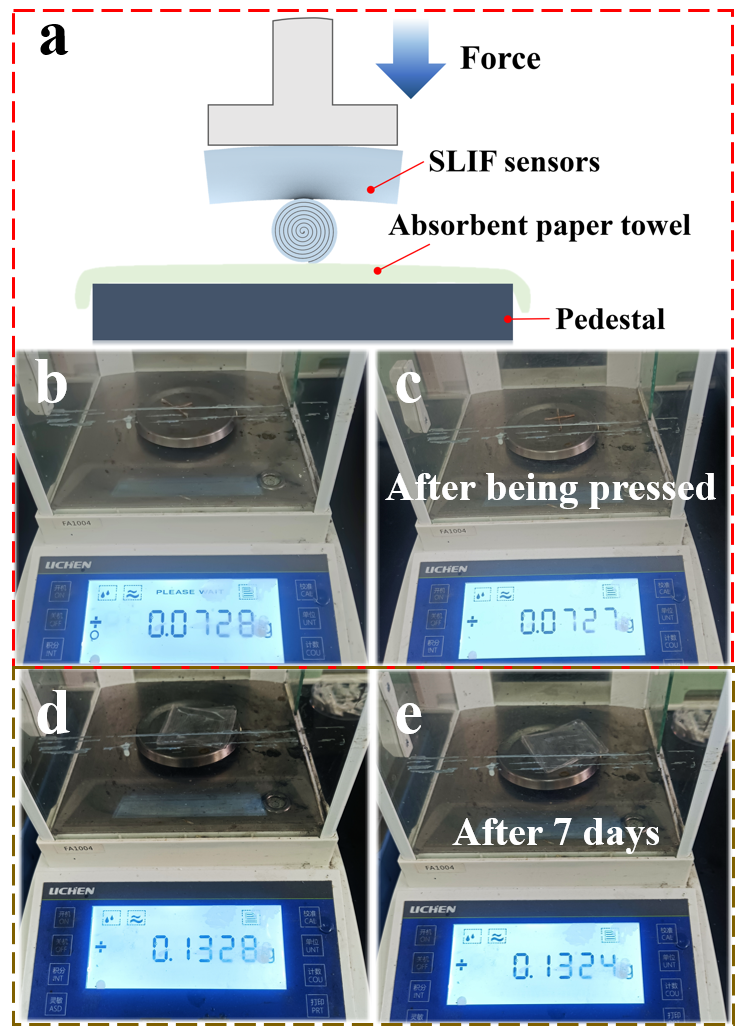


**Figure S2.** **Evaluating the possibility of ion leakage from the SLIF sensors.** a) Schematic diagram of extreme loads (>1 MPa) applied to the SLIF sensors. Weight comparison of SLIF sensors before (b), and after (c) being subjected to extreme load; Weight comparison of TPU/IL film before (d) and after (e) seven days.

In order to evaluate the possibility of ionic leakage in our SLIF sensors, we conducted relevant experiments and the results were presented in Figure S2. In the experiment, we first placed the SLIF sensor on an absorbent paper towel and applied extreme pressure (>1 MPa) to the sensor, as depicted in Figure S2a. If any ionic liquid was leaked from the sensor during this process, the leaked ionic liquid would be absorbed by the towel, resulting in a weight reduction of the sensors. We used an electronic scale to measure the weight change of the SLIF sensor before and after being subjected to extreme pressure. The results show that the weight of the SLIF sensors remained nearly unchanged (Figure S2b-c, from 0.0728 g to 0.0727 g), indicating that our SLIF sensors do not suffer ionic leakage even under extreme pressure.

Additionally, we also tested the possibility of ionic leakage over a long period of time. As shown in the Figure S2d-e, the weight of TPU/IL film remained essentially unchanged after seven days of exposure to air (from 0.1328 g to 0.1324 g), further confirming that our SLIF sensors do not have ionic leakage over time.

The absence of ionic leakage for the SLIF sensors can be attributed to the excellent solubility of the ionic liquid ([EMIM]^+^[TFSI]^-^) within the TPU we used as the substrate,^[10]^ and the low content of ionic liquid has been uniformly dispersed and embedded in the TPU matrix, ensuring a highly stable state. This is also the reason why we choose ionic liquid and TPU as the ionically conducting materials, as they provide better stability than conventional hydrogels.


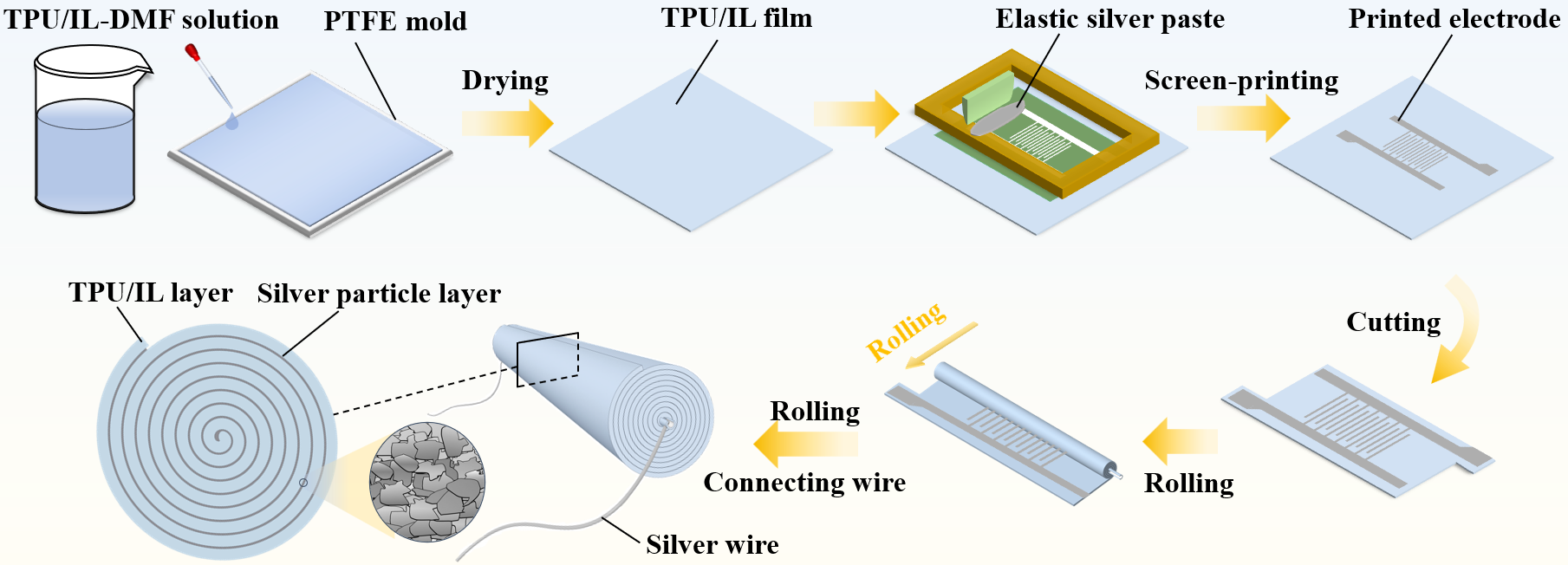


**Figure S3. Schematic illustrations showing the fabrication process of the SLIF sensors.**

TPU/IL composite films (≈80 μm in thickness) were prepared via solution casting method. Two interdigitated electrodes were printed onto the TPU/IL films using elastic silver paste. Then, the TPU/IL films with printed interdigitated Ag electrodes were rolled into SLIF sensors with a unique spirally-layered structure, followed by curing at 80 ℃ for 1 hour to form the final SLIF sensors. It is noteworthy that the self-adhesive properties of the TPU/IL films enable the spiral layers to bond tightly and fuse deeply with each other during the curing process.


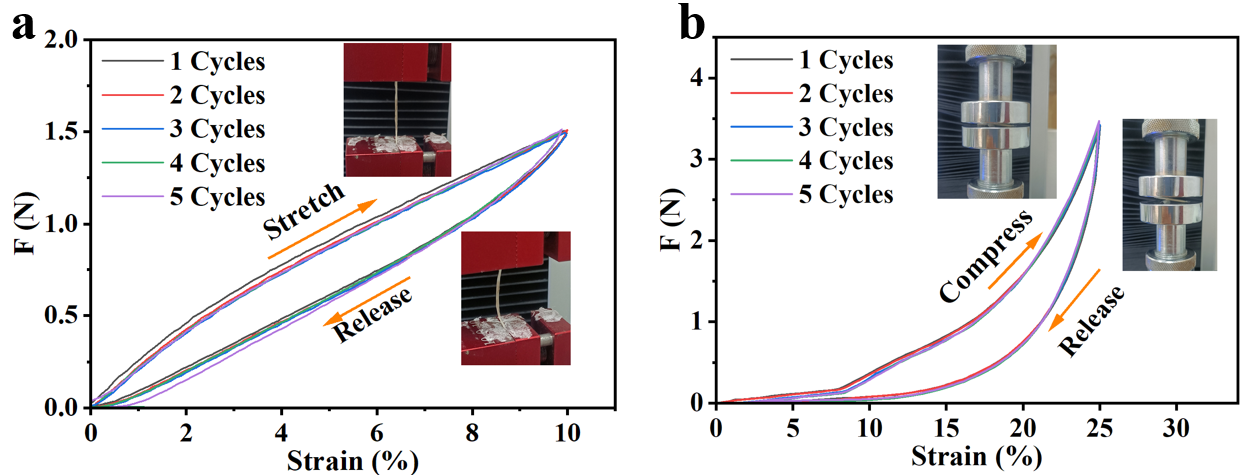


**Figure S4. Mechanical properties test of the SLIF sensors.** a) Stress-strain curve of the SLIF sensor under five stretching cycles. b) Stress-strain curve of two perpendicular SLIF sensors under five compression cycles.

The SLIF sensor was subjected to cyclic tensile test at 10% strain to evaluate its resilience and deformation hysteresis, as shown in Figure S4a. The results reveal the desirable resilience of the SLIF sensor, with slight deformation hysteresis. In addition to good tensile resilience, the SLIF sensors also have good compressive resilience. As a demonstration, the compressive resilience of the SLIF sensor was evaluated with cyclic compressions at 25% strain, as illustrated in Figure S4b. The result indicates desirable compressive resilience of the SLIF sensor. The desirable tensile and compressive resilience give rise to good stability and repeatability of the SLIF sensor.


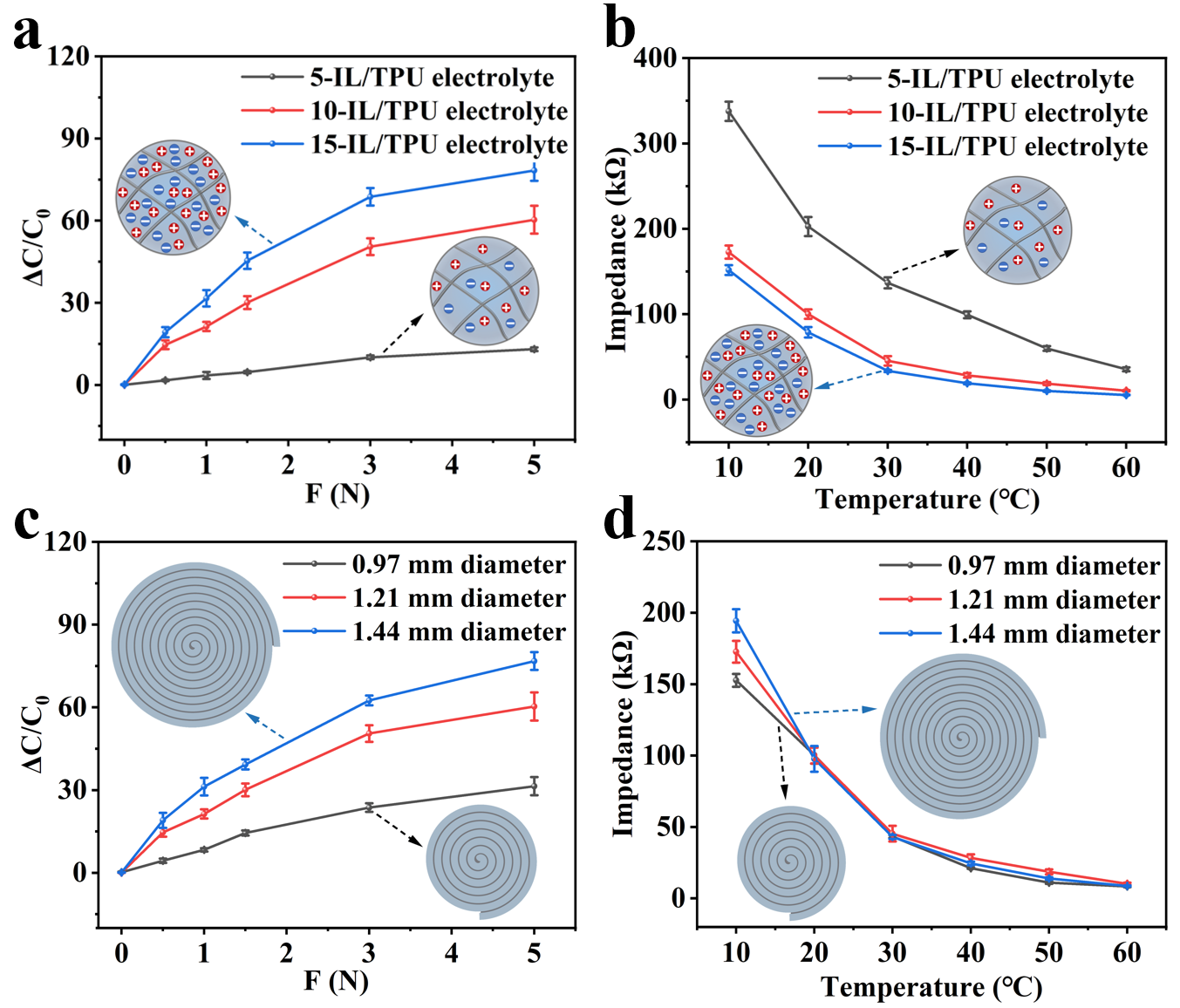


**Figure S5.** **Modulating the mechanical and thermal sensing capabilities of SLIF sensors based on the IL content and diameters of sensors.** a) Mechanical sensing property and (b) thermal sensing property of SLIF sensors with different IL contents (i.e., 5 wt%, 10 wt%, and 15 wt%). c) Mechanical sensing property and (d) thermal sensing property of SLIF sensors with different diameter (i.e., 0.97 mm, 1.21 mm, and 1.44 mm).

According to the Figure S5a, increasing the IL content from 5 wt% to 15 wt% results in an enhancement of the mechanical sensitivity. In contrast, increasing the IL content in the SLIF sensors gives rise to lower thermal sensitivity (Figure S5b). To achieve a good balance between the mechanical and thermal sensing performance, TPU/IL composite with 10 wt % IL was employed to fabricate the SLIF sensors in the following experiment. In addition, the effect of SLIF sensor diameters on the mechanical and thermal sensing performance was also investigated. As shown in Figure S5c-d, larger diameter of sensor leads to higher mechanical sensitivity. On the contrary, the thermal sensing performance is not significantly affected by the sensor diameter. This is due to that thermal sensing relies on the intrinsic ionic impedance variation rather than the geometry of the SLIF sensors.


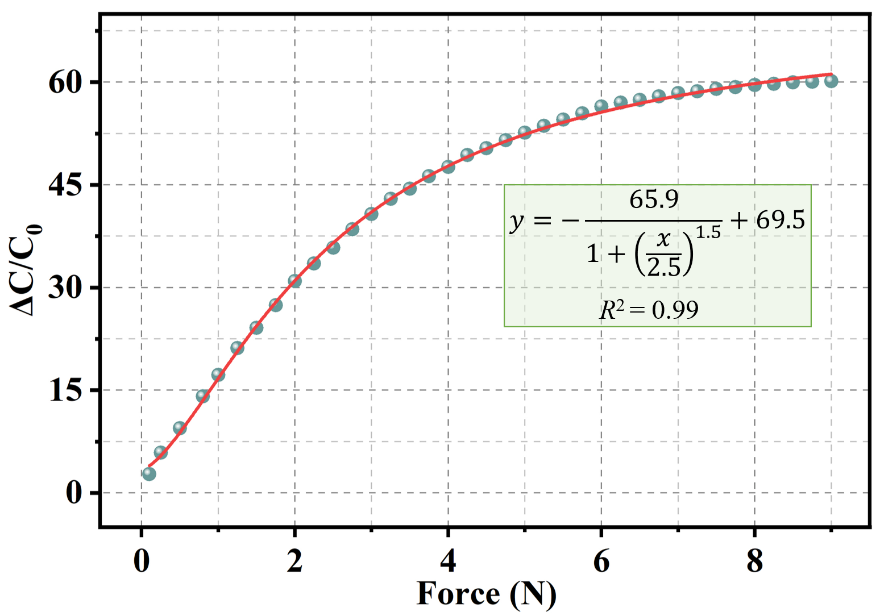


**Figure S6. Experimental results and nonlinear curve fitting of the relationship between the applied force and relative capacitance change (Δ*C*/*C₀*) of the SLIF sensors**.


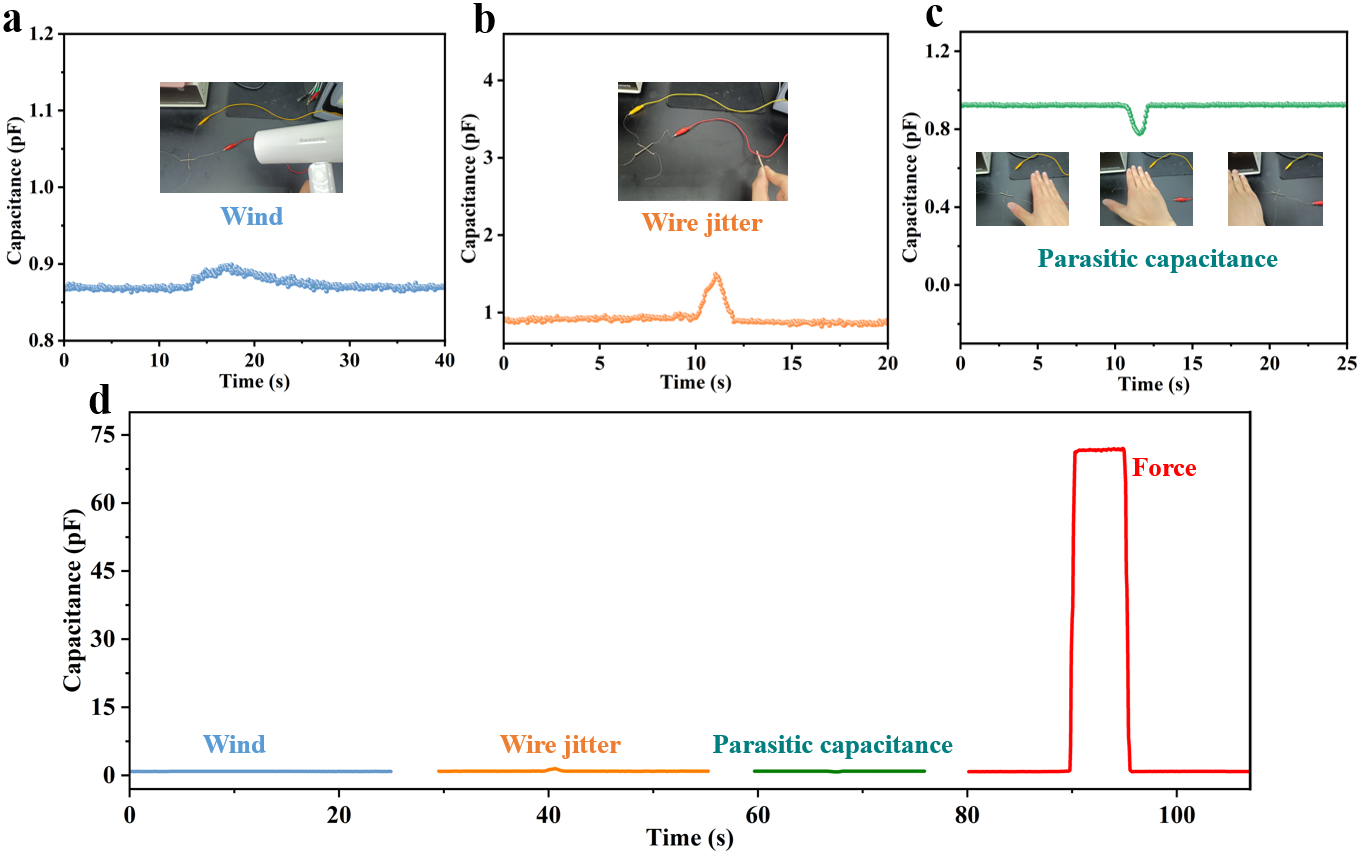


**Figure S7**. **Changes in the capacitance signals of the SLIF sensors under various environmental infl****uences.** a) Wind, b) Wire jitter, c) Parasitic capacitance, d) Applied forces.

We conducted relevant experiments to investigate the influence of environmental disturbance (wind, wire jitter, and parasitic capacitance) on the capacitance value of our SLIF sensors. As shown in the Figure S7a, when we used a hair dryer to blow air to the sensor at a distance of 20 cm, the capacitance signal exhibited only a slight increase (Δ*C* < 0.05 pF), and the baseline experienced a minor change at the end (Δ*C* < 0.02 pF). Therefore, the impact of wind on our SLIF sensors is negligible and can be reasonably disregarded. To simulate the environmental disturbance caused by wire jitter, we utilized a wooden stick to move the wire. As presented in the Figure S7b, the capacitance signal showed an increase during the movement of the wire (from 0.9 pF to 1.5 pF), while the baseline remains essentially unchanged at the end. We also simulated the environmental disturbance of parasitic capacitance by approaching the SLIF sensors with subject’s hand, thereby affecting the sensors with the body capacitance. The results in Figure S7c indicated a slight decrease (from 0.9 pF to 0.75 pF) in the capacitance signal of the sensor as the hand approached, while the baseline remained essentially unchanged at the end.

Although the disturbance of wire jitter and parasitic capacitance have some effects on the sensor capacitance signal, when compared to the capacitance change (Δ*C* > 70 pF) caused by applied forces (Figure S7d), the impact of these small capacitance changes (Δ*C* < 0.6 pF) can be ignored. This is also one reason why the mechanical sensing function is designed to be realized via interfacial ionic supercapacitance variations between two perpendicular SLIF sensors. Compared to traditional pressure sensors based on parallel-plate capacitors, our iontronic pressure sensors exhibit significant variations in capacitance signals under pressure, making them highly resistant to external disturbance.


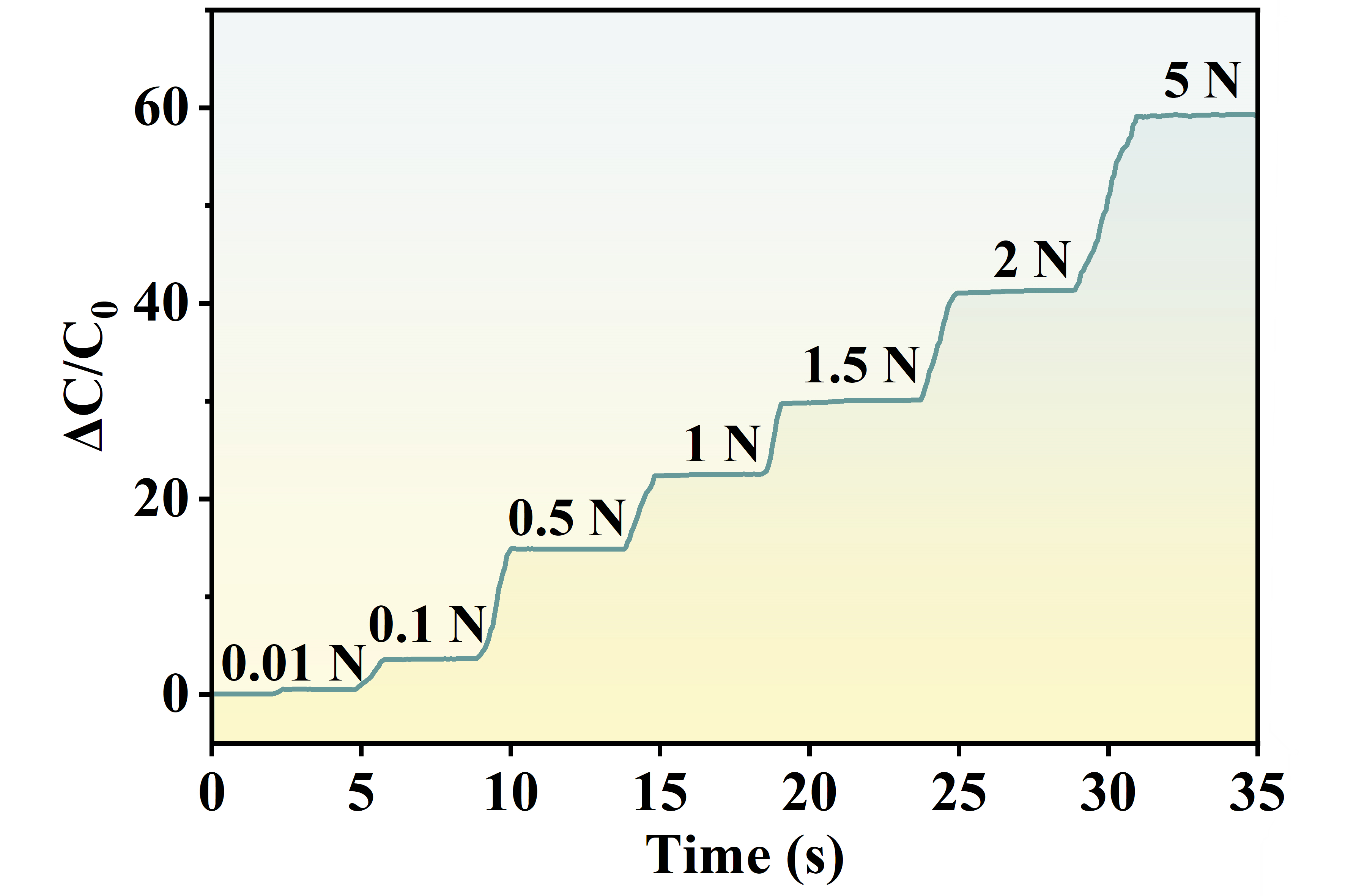


**Figure S8. Response curves of the mechanical sensing units under continuous force variation in a step manner.**


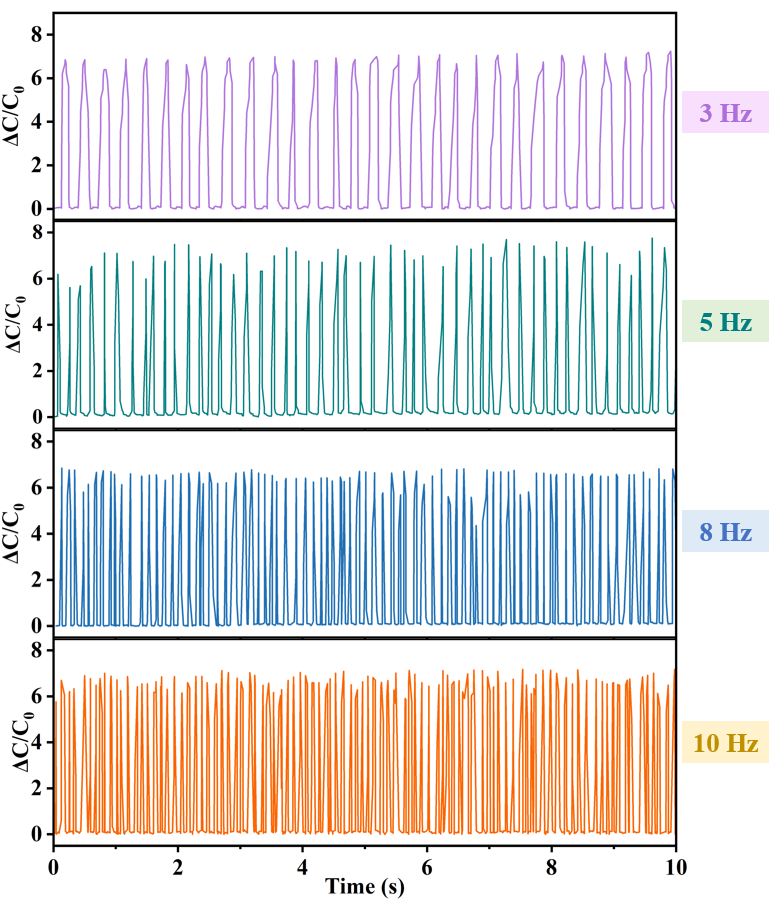


**Figure S9. Response curves of the mechanical sensing units under different frequencies of 3 Hz, 5Hz, 8Hz and 10 Hz.**

As shown in the below Figure S9, the frequency of stimuli within 10 Hz are well distinguished by our SLIF sensors. It is important to clarify that the frequency detection range of 10 Hz is not limited by the sensor's capabilities but by the maximum signal acquisition rate of the signal measurement equipment. Higher frequency stimuli cannot be detected because of the slow signal acquisition rate of the measurement equipment.

Moreover, we have evaluated the accuracy of our sensor's frequency detection capability by comparing the number of signal waves over a given period of time with the expected number of stimuli at the corresponding frequency. The error between the set frequency and the test frequency exhibits a slight deviation of less than 5%, indicating the desirable high frequency detection capability of the sensors.

**
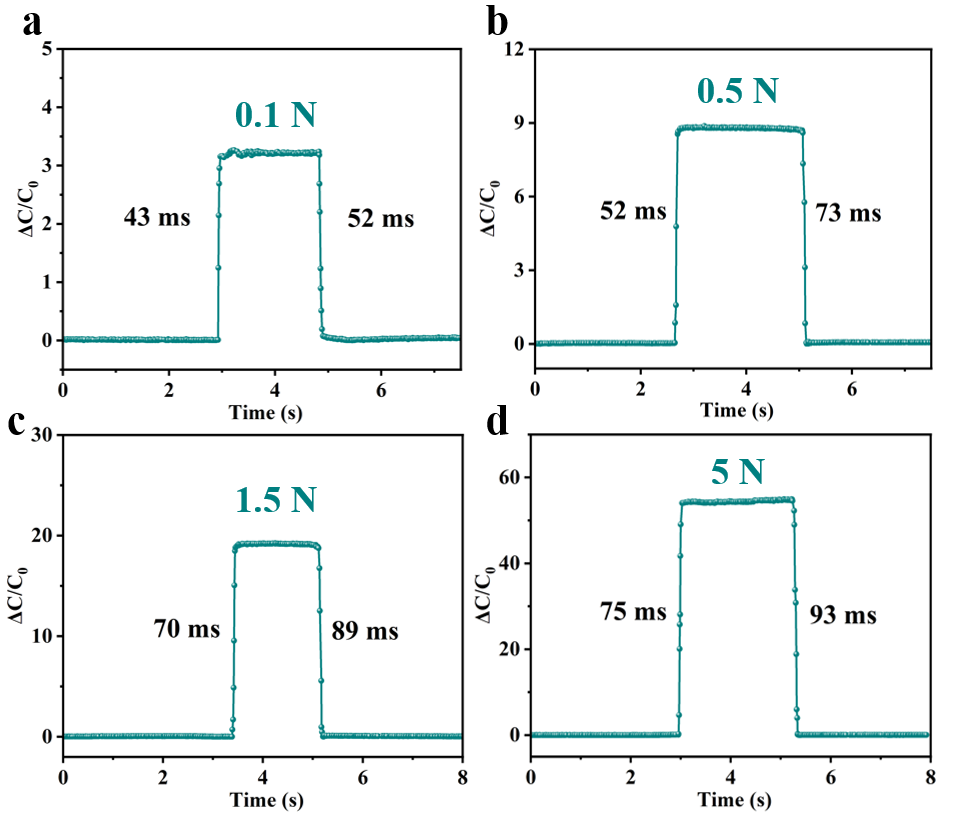
**

**Figure S10. Response and recovery times of the mechanical sensing units under different applied forces.** a) 0.1 N, b) 0.5 N, c) 1.5 N, d) 5 N.

With the increase of applied forces, both the response time and recovery times of mechanical sensing units increase correspondingly. This can be attributed to that larger force causes larger deformation in the SLIF sensors, which subsequently requires more time for the response signal to stabilize. Similarly, the time required for the sensor recovery from the deformed state to the original state also increases with higher applied forces. However, the increase rate of response/recovery times gradually slows down with increased forces. Since there is an upper limit for the deformation of the SLIF sensors under force, causing the response/recovery times to reach a saturation value.


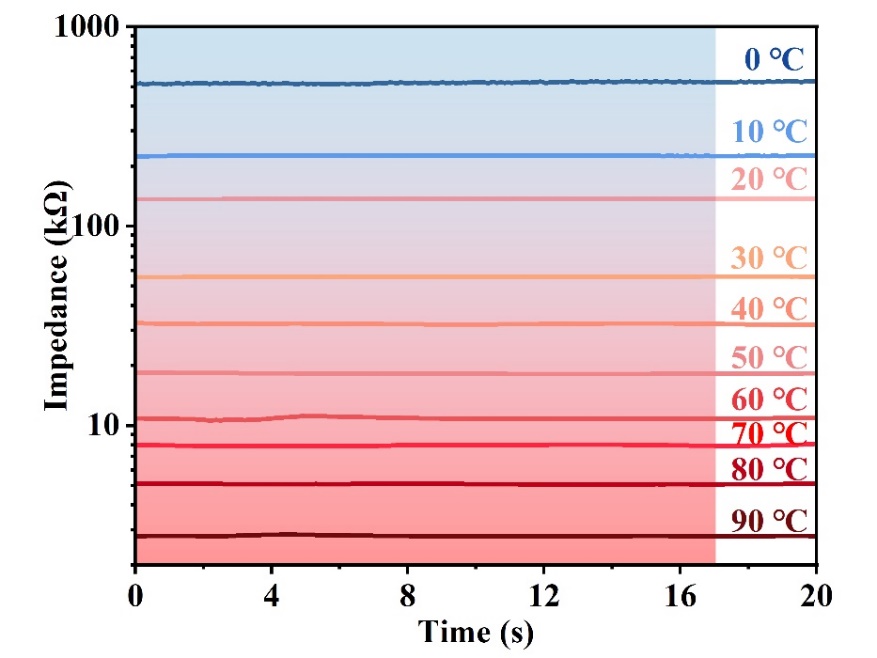


**Figure S11.** **Corresponding impedance of the thermal sensing units under different temperatures in the range of 0-90 ℃ at intervals of 10 ℃.**


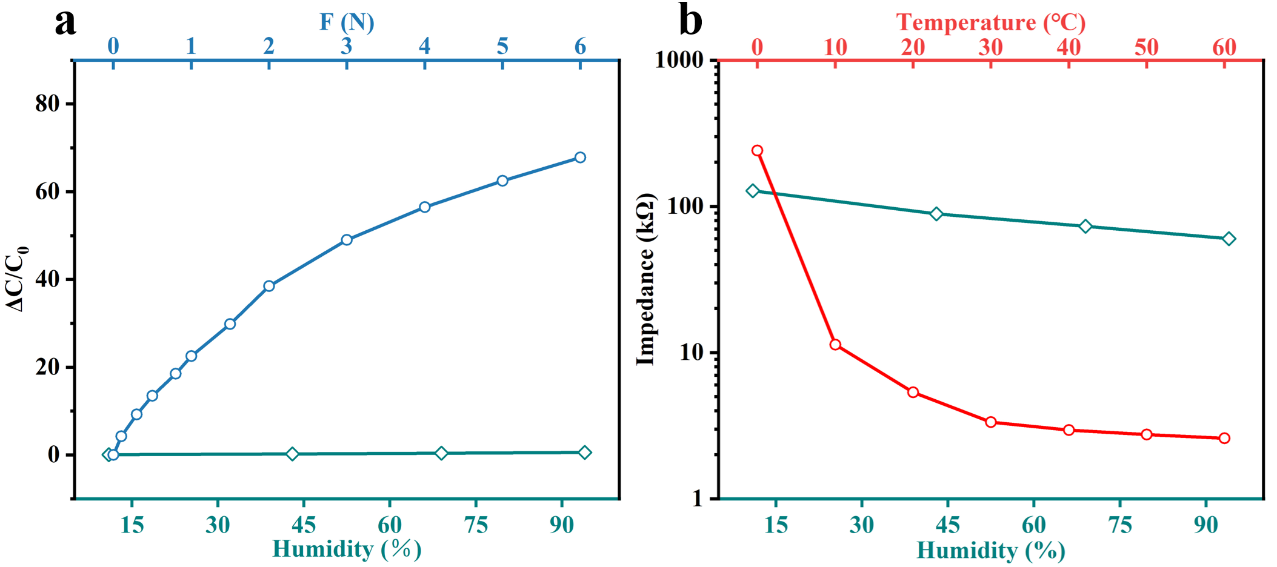


**Figure S12.** **Effect of humidity variations on the mechanical and thermal sensing performance of the SLIF sensors.** a) Relative capacitance change of the mechanical sensing units under different forces and humidities. b) Impedance change of the thermal sensing units under different temperatures and humidities.

To evaluate the effect of humidity on the performance of the SLIF sensors, sensors were placed in targeted humidity environment for over eight hours, followed by measuring the mechanical and thermal sensing performance. The experiment results indicate that changes in relative capacitance change (Δ*C*/*C₀*) and impedance (*Z*) caused by force and temperature variation are much higher than that caused by humidity variation. For instance, when the humidity increased from 11% to 94%, the variation in Δ*C*/*C₀* is only 0.6 (Figure S12a), which is much lower than that by applying a force of 0 to 7 N (Δ*C*/*C₀* ≈ 67). Similarly, the ionic impedance decreased from 128 kΩ to 60 kΩ (only 68 kΩ variation) under the increased humidity from 11% to 94% (Figure S12b), which is much smaller than that by increasing the temperature from 0 ℃ to 90 ℃ (524 kΩ variation). These results can be attributed the hydrophobicity of TPU/IL matrix and the spiral structure of SLIF sensors, which effectively minimize the effects of external humidity on the sensing performances of SLIF sensos.


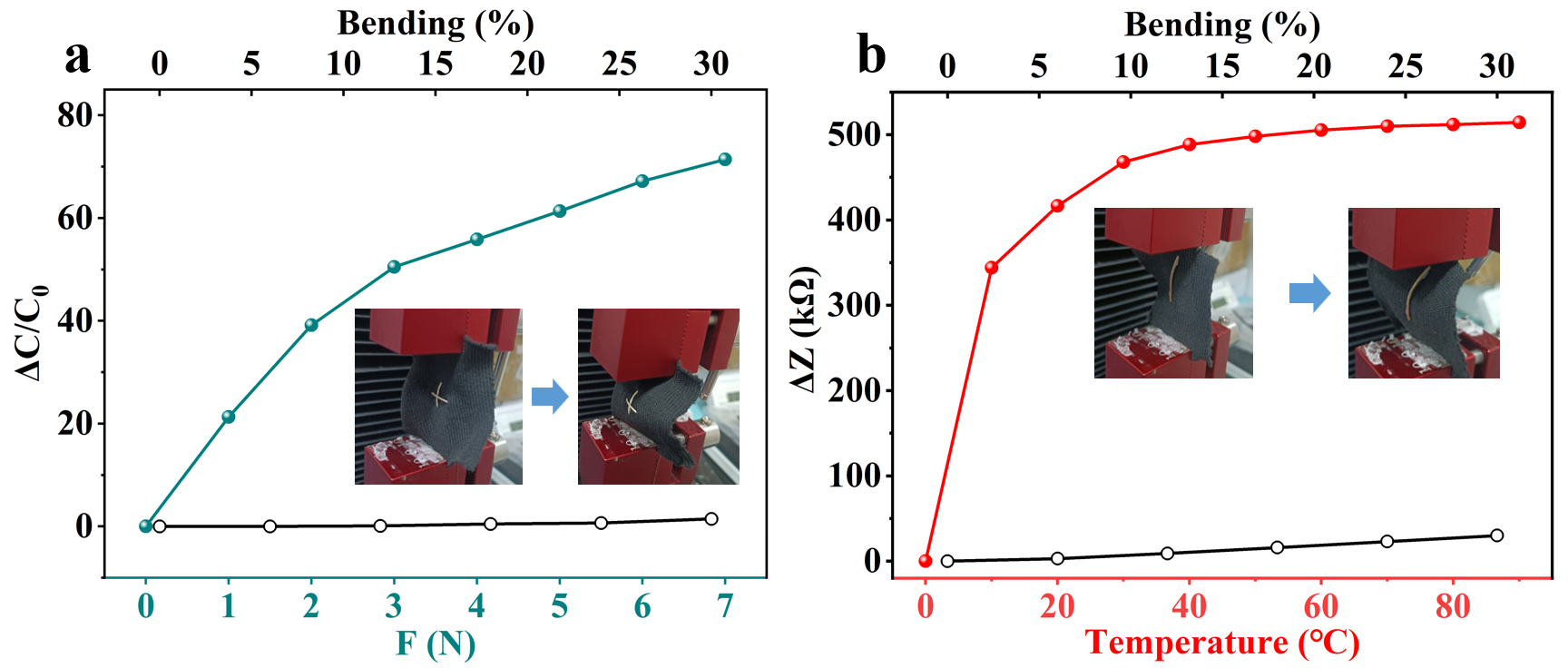


**Figure S13.** **Impact of fabric bending on the stability of SLIF sensors integrated in fabric.** a) Mechanical sensing mode. b) Temperature sensing mode.

As illustrated in Figure S13, with the increasing of bending degree of the fabric, the capacitance signal of the SLIF sensor integrated within the fabric exhibits a slight increase (Figure S13a), while the impedance shows a slight decrease (Figure S13b). At the maximum bending degree of the fabric (30%, bending distance: 3 cm), the increment of relative capacitance change (Δ*C*/*C_0_*) signal is just 1.5, and the impedance change (Δ*Z*) signal is approximately 30 kΩ. However, these signal variations caused by fabric deformation are far smaller and negligible when compared to the signal changes caused by mechanical stimulus (Δ*C*/*C_0_* > 70) and temperature stimulus (Δ*Z* > 500 kΩ). In conclusion, although the stability of our SLIF sensors may be affected by the fabric deformation, it does not significantly compromise the reliability of the proposed sensors.


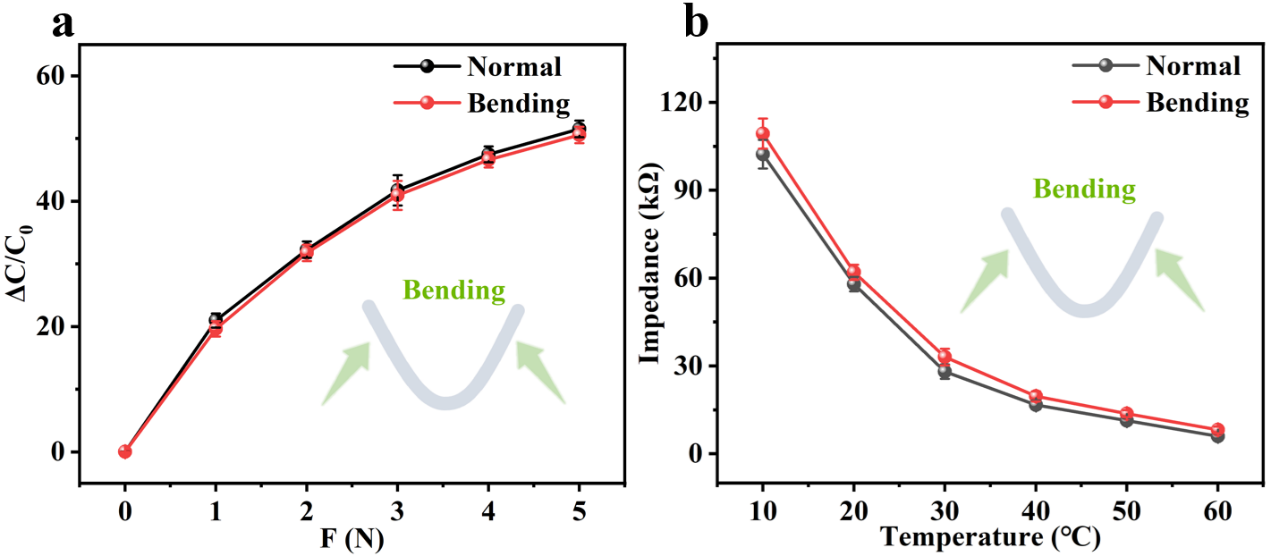


**Figure S14. Comparison of mechanical sensing performance (a) and thermal sensing performance (b) of SLIF sensors before bending (normal) and after 100 cycles of bending (bending radius: 2 cm).**

The interdigitated electrodes are fully embedded in (rather than attached on) the SLIF sensors, which endows the SLIF sensors with superior robustness compared with conventional electronic fibers or textiles. Compared with the normal SLIF sensors, the mechanical and thermal sensing performance of sensors after 100 cycles of bending exhibit negligible change in their sensing performance, showing good mechanical robustness.


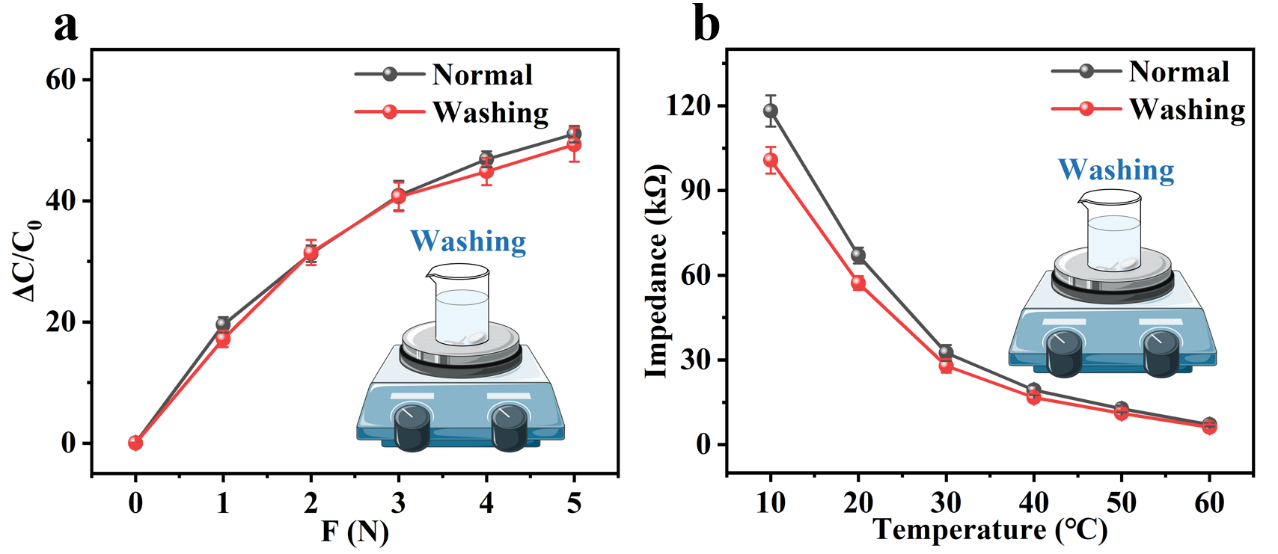


**Figure S15. Comparison of mechanical sensing performance (a) and thermal sensing performance (b) of SLIF sensors before washing (Normal) and after washing in a 1% detergent solution for 1h at 500 rpm.**

Compared with the normal SLIF sensors, after washing in a 1% detergent solution for 1h at 500 rpm mechanical stirring, the thermal sensing performance of sensors exhibit a slight variation, while the mechanical performance is invariable. These results demonstrate the good resistance of the SLIF sensors to washing treatment.


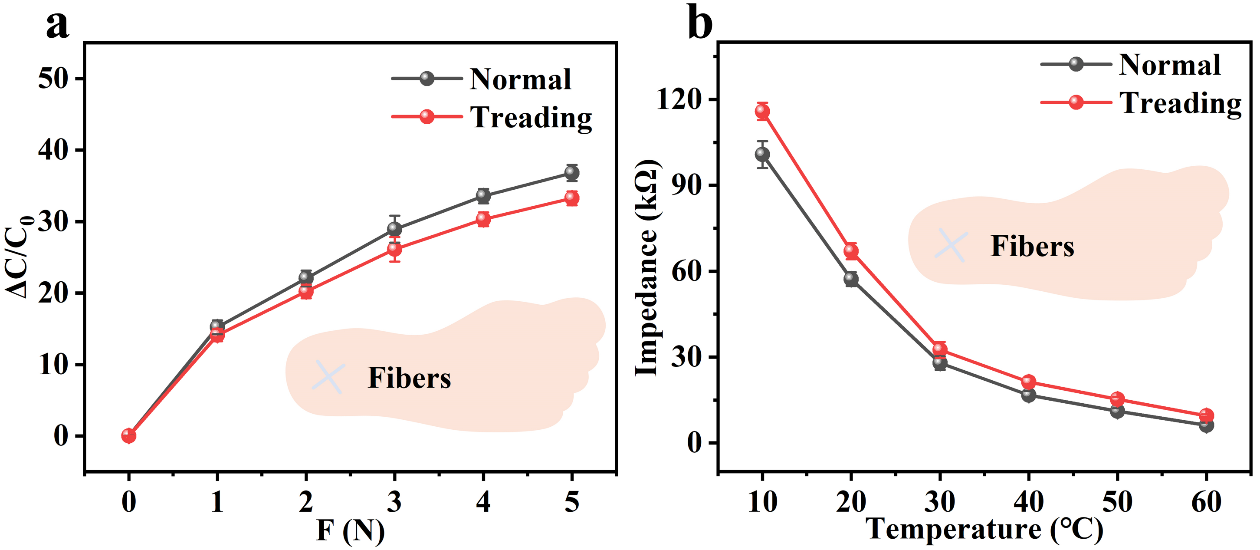


**Figure S16. Comparison of mechanical sensing performance (a) and thermal sensing performance (b) of SLIF sensors before (normal) and after undergoing 300 cycles of compressing with the heel of a subject of 74 kg (****Treading).**

The mechanical and thermal sensing performance of sensors exhibit slight change before and after undergoing 300 mechanical compressing cycles of over 360 N (i.e., strike the SLIF sensors with the heel of a subject of 74 kg).


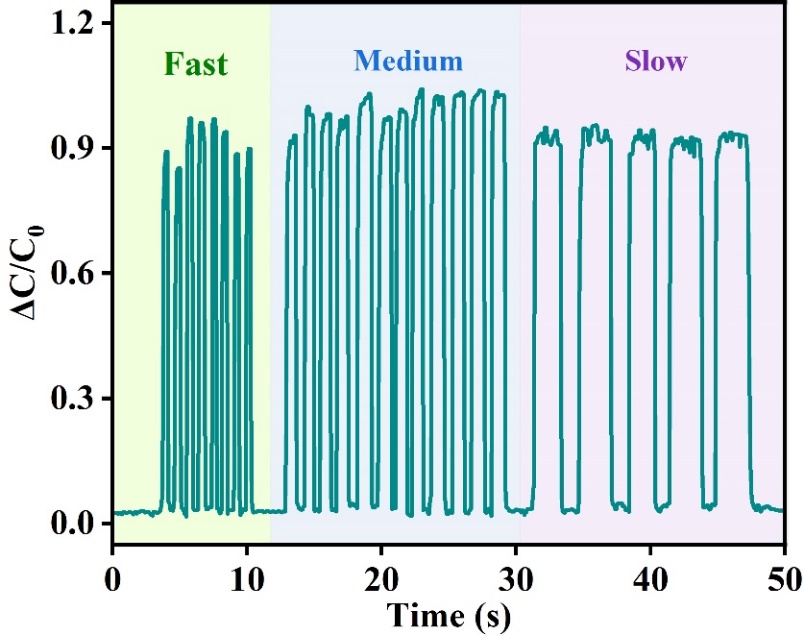


**Figure S17. Response signals of** **a SLIF sensor-innervated smart mask to** **an air flow that is similar to normal breathing.**

Each exhalation cycle would cause a subtle air pressure variation to the mask. To verify the ability of the smart mask in detecting subtle respiratory air pressure variation, a similar air pressure was applied to the smart mask using a commercial airbrush. From the results, it is noticed that the subtle respiratory air pressure can be well detected by the SLIF sensor-innervated smart mask.


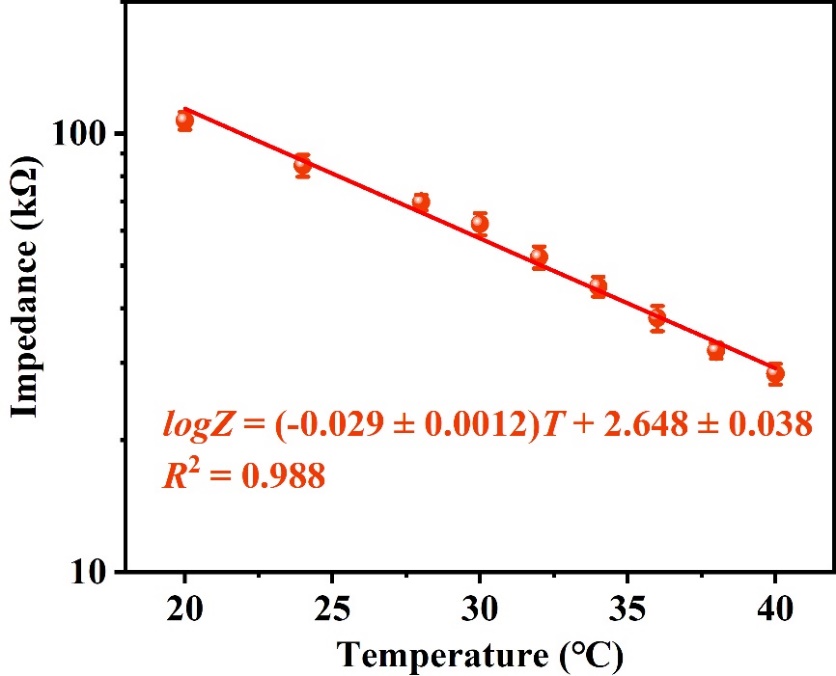


**Figure S18.** **Experimental results and fitting curve of the relationship between the temperature value and the impedance value of the SLIF sensor-innervated smart mask.**

From the above temperature calibration curve of the thermal sensors, the measured impedance value could be converted into temperature value.


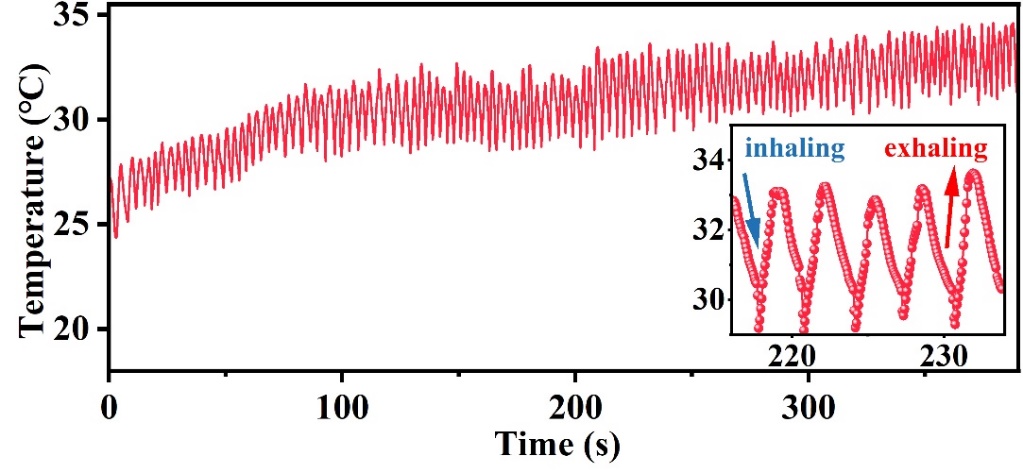


**Figure S19. Real-time temperature changes during the respiratory process.**

The exhalation process causes the temperature to rise slightly inside the mask, while the inhalation process causes the temperature to decrease slightly. During a prolonged respiratory test, the heat accumulated by warm air exhaled from the lungs gradually increase the temperature inside the mask.


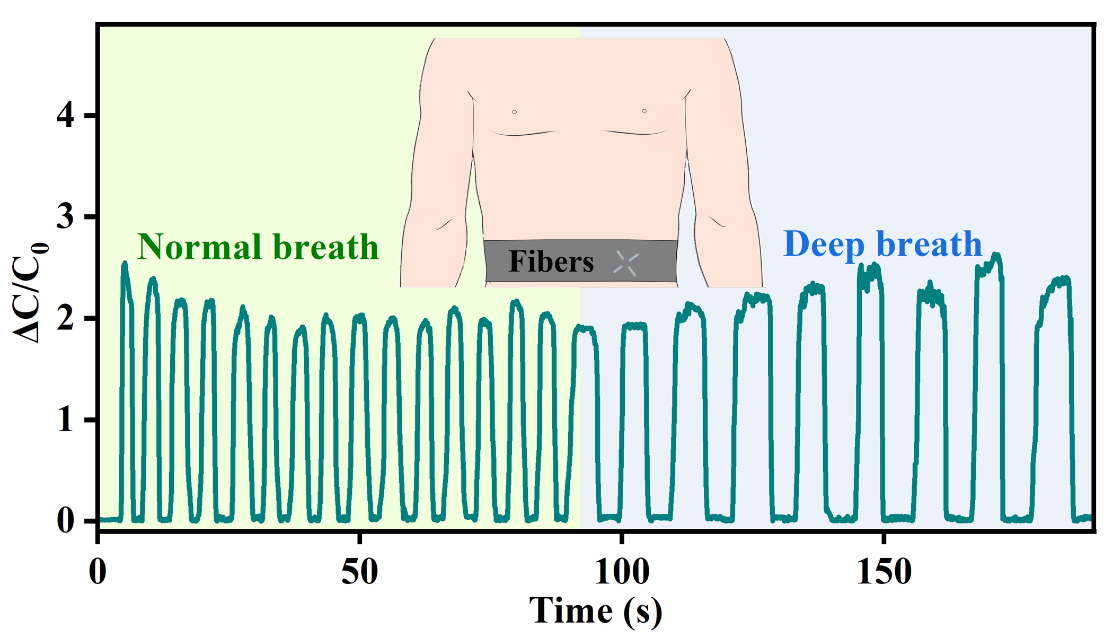


**Figure S20. Monitoring of respiration situations (normal breath and deep breath) using a** **SLIF sensor-innervated smart belt that is fixed to the abdomen.**

Along with the breathing rhythm, the abdomen cavity expands in the inspiration process and contracts during expiration. Continuous monitoring of respiration can be achieved by tracking the periodic movement of the abdomen cavity with smart elastic blet innervated with SLIF sensors.


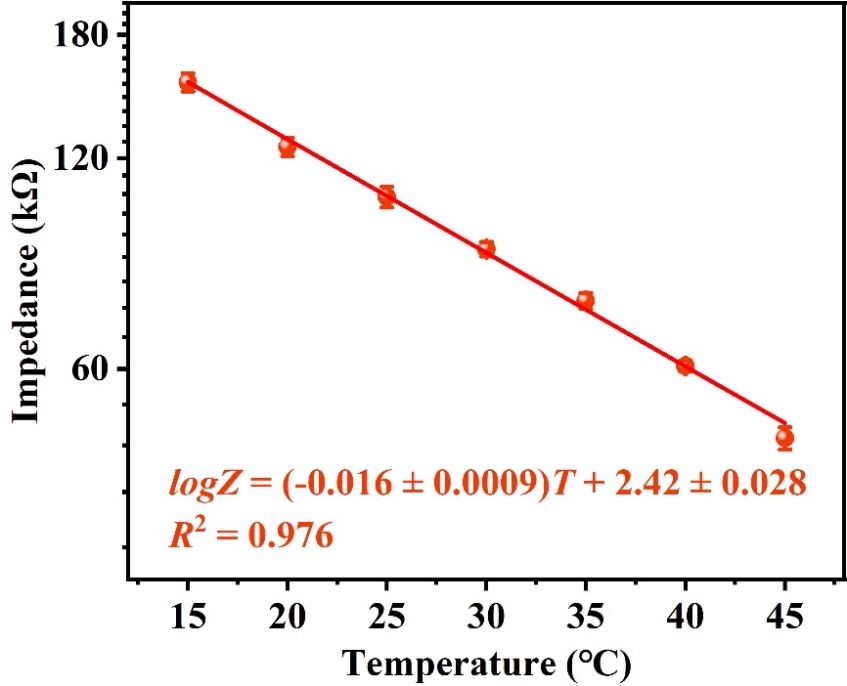


**Figure S21. Experimental results and fitting curve of the relationship between the temperature value and the impedance value of the SLIF sensor-innervated smart wrist band.**

From the above temperature calibration curve of the thermal sensors, the measured impedance value could be converted into temperature value.

**
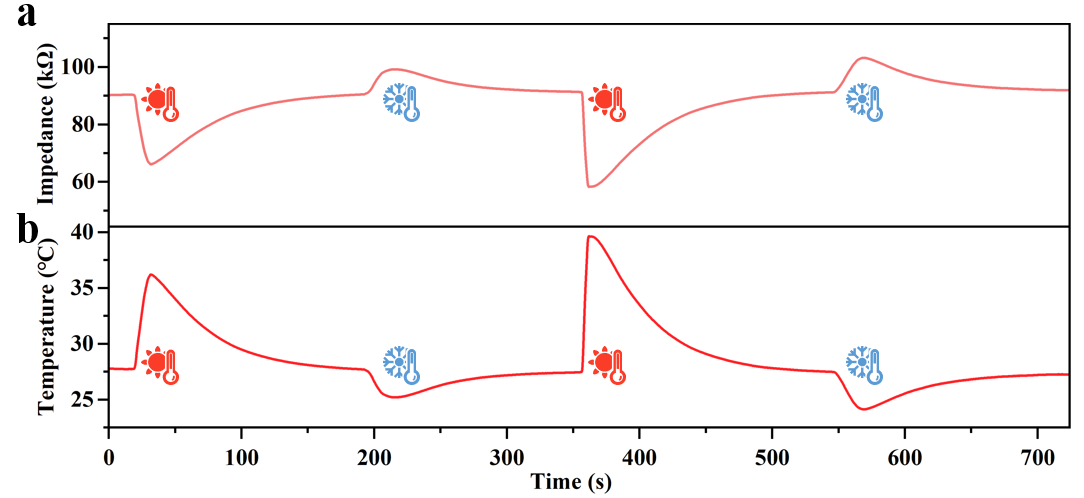
**

**Figure S22.** **Temperature monitoring on skin surface based on a SLIF sensor-innervated smart wrist band.** (a) Impedance value changes of the SLIF sensor-innervated smart wrist band when changing the skin temperature of the wrist part. (b) Calculated temperature changes of the wrist part.


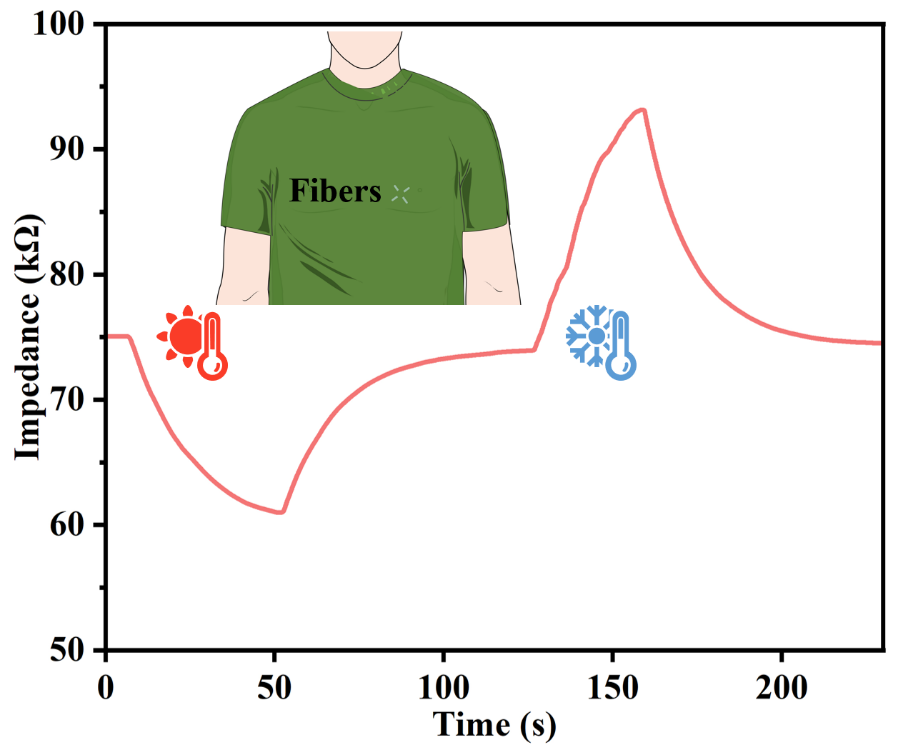


**Figure S23. Skin temperature monitoring based on a SLIF sensor-innervated smart T-shirt.**


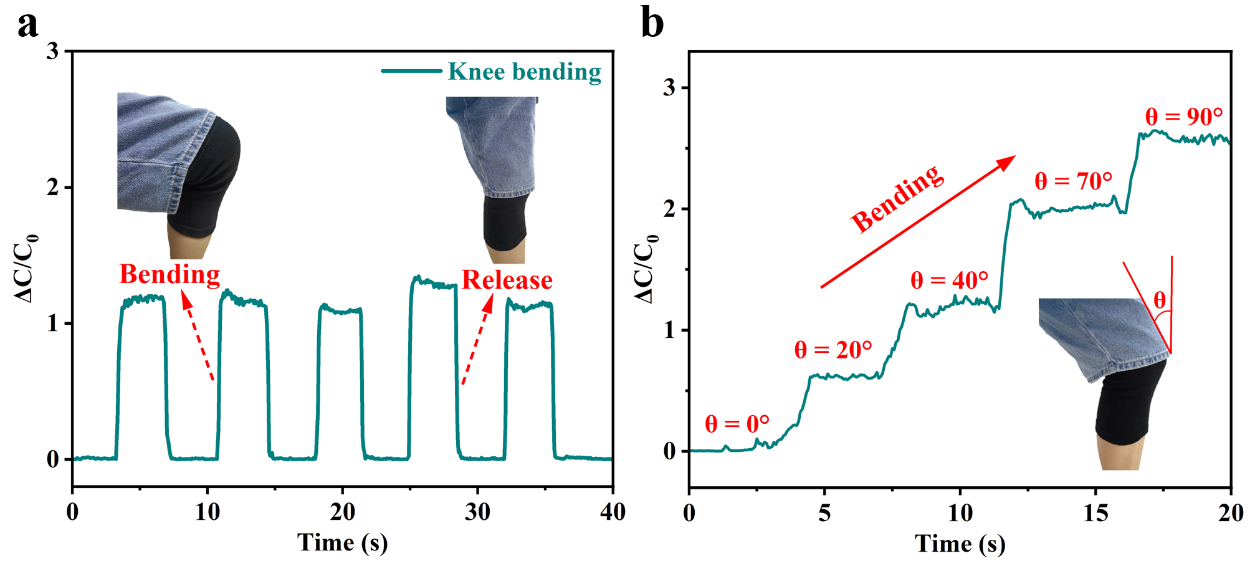


**Figure S24.** **Knee motions monitoring using the** **smart kneelet innervated with the SLIF sensors.**

Bending motions of knee can generate a mechanical force, leading to a rapid rise in the relative capacitance change signals of the SLIF sensor sensors (Figure S24a). As the bending angle increases, the squeeze on the SLIF sensors by the smart kneelet is enhanced, resulting the increase of the relative capacitance change (Figure S24b).


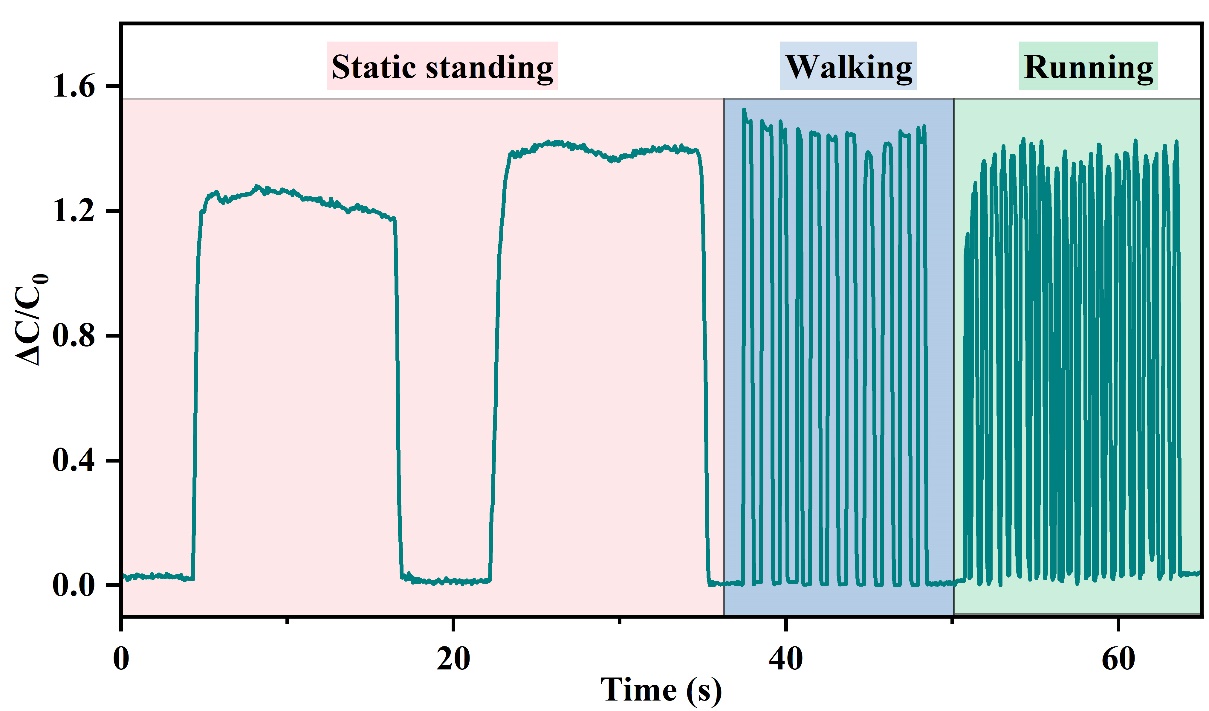


**Figure S25.** **Monitoring of the walking states (i.e., static standing, walking, and running) based on the smart socks innervated with SLIF sensors.**

When the subject wearing the smart sock innervated with SLIF sensors stands still, the foot force measured by the smart sock is constant. During walking and running, the recorded signals show good repeatability and low hysteresis.

**
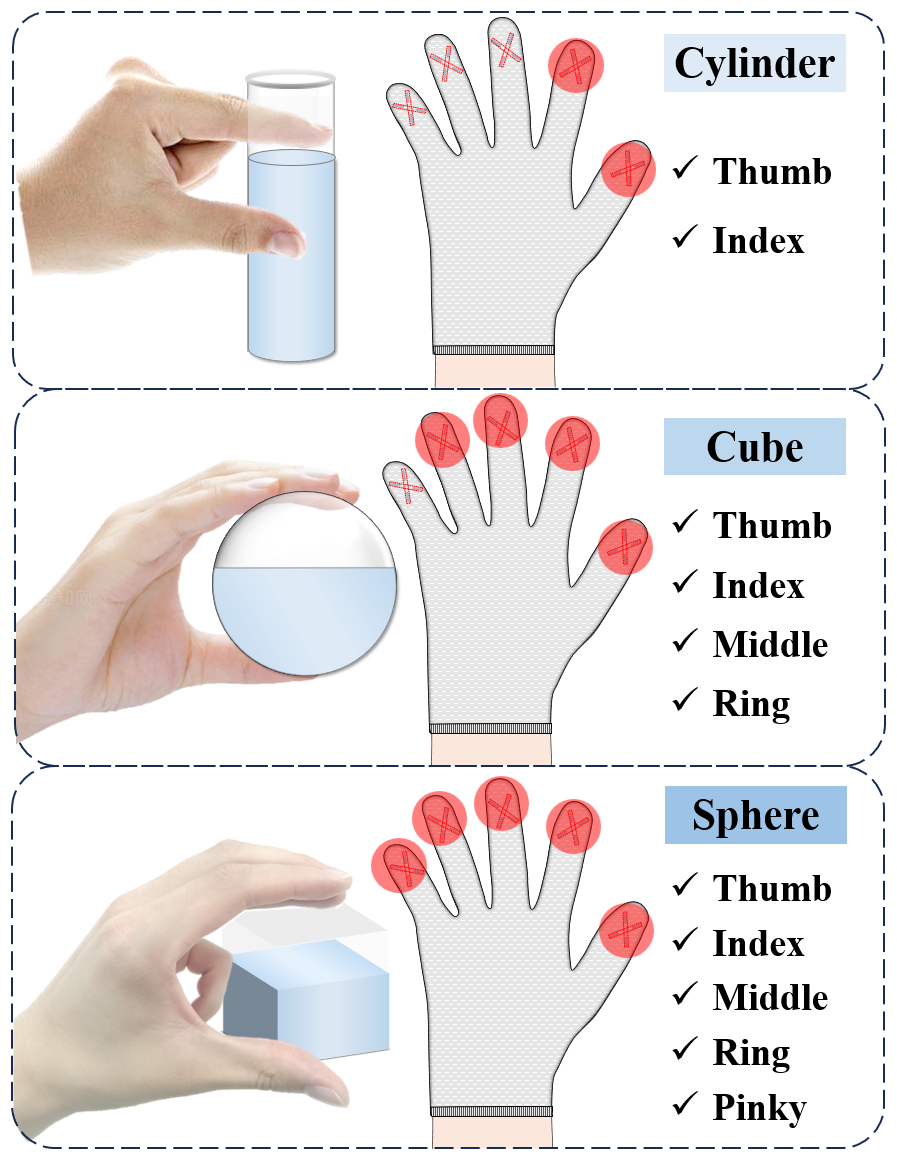
**

**Figure S26. Schematic diagram of activating different mechanical sensing units with different shaped objects.**

**Supplementary Reference**

[1] H. Wang, S. Li, Y. Wang, H. Wang, X. Shen, M. Zhang, H. Lu, M. He, Y. Zhang, *Adv. Mater.* **2020**, 32, 1908214.

[2] C. Wang, X. Li, E. Gao, M. Jian, K. Xia, Q. Wang, Z. Xu, T. Ren, Y. Zhang, *Adv. Mater.* **2016**, 28, 6640.

[3] M. Clevenger, H. Kim, H. W. Song, K. No, S. Lee, *Sci. Adv.* **2021**, 7, eabj8958.

[4] T. Chang, S. Akin, M. K. Kim, L. Murray, B. Kim, S. Cho, S. Huh, S. Teke, L. Couetil, M. B.-G. Jun, C. H. Lee, *Adv. Mater.* **2022**, 34, 2108021.

[5] Y. Wu, S. S. Mechael, C. Lerma, R. S. Carmichael, T. B. Carmichael, *Matter* **2020**, 2, 882.

[6] Q. Liu, Y. Zhang, X. Sun, C. Liang, Y. Han, X. Wu, Z. Wang, *Chem. Eng. J.* **2023**, 454, 140302.

[7] Y. Luo, Y. Li, P. Sharma, W. Shou, K. Wu, M. Foshey, B. Li, T. Palacios, A. Torralba, W. J. N. E. Matusik, *Nat. Electron.* **2021**, 4, 193

[8] J. Gao, Y. Fan, Q. Zhang, L. Luo, X. Hu, Y. Li, J. Song, H. Jiang, X. Gao, L. Zheng, W. Zhao, Z. Wang, W. Ai, Y. Wei, Q. Lu, M. Xu, Y. Wang, W. Song, X. Wang, W. Huang, *Adv. Mater.* **2022**, 34, 2107511.

[9] H. Zhai, L. Xu, Z. Liu, L. Jin, Y. Yi, J. Zhang, Y. Fan, D. Cheng, J. Li, X. Liu, Q. Song, P. Yue, Y. Li, *Chem. Eng. J.* **2022**, 439, 135502.

[10] a)M. L. Jin, S. Park, Y. Lee, J. H. Lee, J. Chung, J. S. Kim, J.-S. Kim, S. Y. Kim, E. Jee, D. W. Kim, J. W. Chung, S. G. Lee, D. Choi, H.-T. Jung, D. H. Kim, *Adv. Mater.* **2017**, 29; b)S. Y. Kim, Y. Kim, C. Cho, H. Choi, H. W. Park, D. Lee, E. Heo, S. Park, H. Lee, D. H. Kim, *ACS Appl. Mater. Interfaces.* **2019**, 11, 29350; c)C. Chen, W. B. Ying, J. Li, Z. Kong, F. Li, H. Hu, Y. Tian, D. H. Kim, R. Zhang, J. Zhu, *Adv. Funct. Mater.* **2022**, 32, 2106341.
